# Reversible Activation and Transfer of White Phosphorus by Silyl‐Stannylene

**DOI:** 10.1002/anie.202013423

**Published:** 2020-12-21

**Authors:** Debotra Sarkar, Catherine Weetman, Dominik Munz, Shigeyoshi Inoue

**Affiliations:** ^1^ Department of Chemistry, WACKER-Institute of Silicon Chemistry and Catalysis Research Center Technische Universität München Lichtenbergstraße 4 85748 Garching Germany; ^2^ Department of Pure and Applied Chemistry University of Strathclyde Glasgow G1 1XL UK; ^3^ Department of Chemistry and Pharmacy General and Inorganic Chemistry Friedrich-Alexander-University Erlangen-Nuremberg (FAU) Egerlandstraße 1 91058 Erlangen Germany; ^4^ Inorganic Chemistry: Coordination Chemistry Saarland University, Geb. C4.1 66123 Saarbrücken Germany

**Keywords:** main group, P_4_ activation, reversible activation, small molecule activation, tetrylenes

## Abstract

Use of a silyl supported stannylene (^Mes^TerSn(Si^*t*^Bu_3_) [^Mes^Ter=2,6‐(2,4,6‐Me_3_C_6_H_2_)_2_C_6_H_3_] enables activation of white phosphorus under mild conditions, which is reversible under UV light. The reaction of a silylene chloride with the activated P_4_ complex results in facile P‐atom transfer. The computational analysis rationalizes the electronic features and high reactivity of the heteroleptic silyl‐substituted stannylene in contrast to the previously reported bis(aryl)stannylene.

## Graphical abstract

A heteroleptic silyl supported stannylene enables activation of white phosphorus and use as P‐atom transfer reagent.

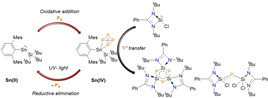

Organophosphorus compounds offer unique structural and electronic properties and therefore have gained considerable attention in the past decades.^[1]^ Their synthesis classically involves the energy intensive and hazardous chlorination of white phosphorus (P_4_).[^1d^, ^1e^] As an alternative, the activation of P_4_ under mild conditions and subsequent transformation to organic molecules presents an attractive approach towards P_4_ utilization.[^1a^, ^1b^, ^1c^, ^1e^, ^1f^] To date, a plethora of examples for transition metal mediated P_4_ activation has been achieved.[^1b^, ^1e^] Very recently, in an elegant study Wolf and co‐workers demonstrated the photocatalytic transformation of P_4_ to aryl phosphines and phosphonium salts by use of an iridium catalyst.^[2]^ In contrast to transition metals, activation of P_4_ with main group elements is limited to only a handful of examples,[^1a^, ^1c^, ^1f^, ^3^] and catalytic utilization of P_4_ with main group compounds remains so far elusive.

Recently, low valent heavier group 14 carbene homologues, namely tetrylenes ([R_2_E:] E=Si, Ge), which are in the +II oxidation state, have given new impetus to the field of P_4_ activation.^[4]^ Examples of both silylenes[^4a^, ^4c^, ^4d^, ^4f^, ^5^] and germylenes have been reported to activate P_4_.^[4b]^ Notably, a diaryl germylene [^Mes^Ter_2_Ge:], (^Mes^Ter=2,6‐(2,4,6‐Me_3_C_6_H_2_)_2_C_6_H_3_] provided the first main group mediated reversible activation of the P−P bond in P_4_.^[4b]^ This is an important discovery, as reversibility is a key step towards main group mediated catalysis.^[6]^ To the best of our knowledge, there is only one example of a dimeric, low‐valent Sn^I^ complex for the controlled activation of P_4_ (Scheme 1 a), whereas such reactivity is unknown for the heavier tetrylene analog, stannylene [R_2_Sn:].^[7]^

**Scheme 1:**
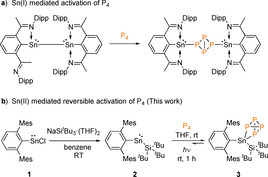
Activation of P_4_ with low‐valent tin complexes (Dipp=2,6‐^*i*^Pr_2_(C_6_H_3_), Mes=2,4,6‐Me_3_(C_6_H_2_).

Stannylenes present themselves as ideal candidates for bond activation and catalysis, due to the increased stability of the +II oxidation state compared to the lighter group 14 tetrylenes.^[8]^ It has previously been shown that the Sn^II^‐Sn^IV^ redox couple can be manipulated by use of strongly σ‐donating boryl ligands to enable dihydrogen activation.^[8c]^ Therefore, we postulated that use of electropositive silyl groups, which have been widely employed as stabilizing ligands in low valent main group chemistry,^[9]^ may enable the activation of strong bonds.

Our study began with the targeted isolation of the sterically demanding homoleptic bis(silyl)stannylene [(Si^*t*^Bu_3_)_2_Sn]. However, various attempts to isolate [(Si^*t*^Bu_3_)_2_Sn] was unsuccessful in our hands. Thus, our attention turned to the synthesis of a heteroleptic silyl substituted stannylene. The heteroleptic silyl stannylene **2** [^Mes^TerSn(Si^*t*^Bu_3_)] was isolated via treatment of chlorostannylene **1** [^Mes^TerSnCl) with one equivalent of NaSi^*t*^Bu_3_⋅(THF)_2_. Compound **2** was isolated in 80 % yield as a dark blue solid (Scheme 1 b) and is soluble in polar solvents such as tetrahydrofuran, but poorly soluble in nonpolar organic solvents such as benzene or toluene. The ^119^Sn{^1^H} NMR spectrum of compound **2** showed a characteristic signal for the tin center at *δ* 197 ppm, which is significantly upfield in comparison to **1** (562 ppm) and the bis(aryl)stannylene (^Mes^Ter)_2_Sn (1971 ppm).[^8e^, ^10^] This indicates an electron rich Sn^II^ center, which can be attributed to the substituent effect (Si^*t*^Bu_3_ vs. ^Mes^Ter). In the ^29^Si NMR spectrum a distinct signal was observed at 94.7 ppm for the silicon atom of Si^*t*^Bu_3_. The calculation of NMR shifts for heavy elements represents a challenge due to spin‐orbit coupling effects and limitations of common basis sets/ effective core potentials (ECP).^[11]^ Nevertheless, calculations at the PBE0‐D3/def2‐TZVPP//PBE0‐D3/def2‐SVP level of theory with the def2‐ECP reproduce the order of the ^119^Sn NMR shifts (**1**: −170 ppm; **2**: −314 ppm; (^Mes^Ter)_2_Sn: 664 ppm) and hence corroborate a comparably electron rich Sn^II^ atom in **1**.

Single crystal X‐ray diffraction (SC‐XRD) analysis confirmed the identity of compound **2**, with the two‐coordinate Sn center bound by one Si^*t*^Bu_3_ and *m*‐terphenyl group (Figure 1). The ∡ C1‐Sn1‐Si1 bond angle in **2** is 113.50(14)° and falls within the range of aryl substituted two‐coordinate Sn^II^ complexes (96.9–117.6°).^[12]^ Notably, two‐coordinate stannylenes [R_2_Sn] with broad bond angles (∡ R‐Sn‐*R*=112 to 118°) have been shown to activate a wide range of small molecules.[^8c^, ^8d^, ^8e^]

**Figure 1:**
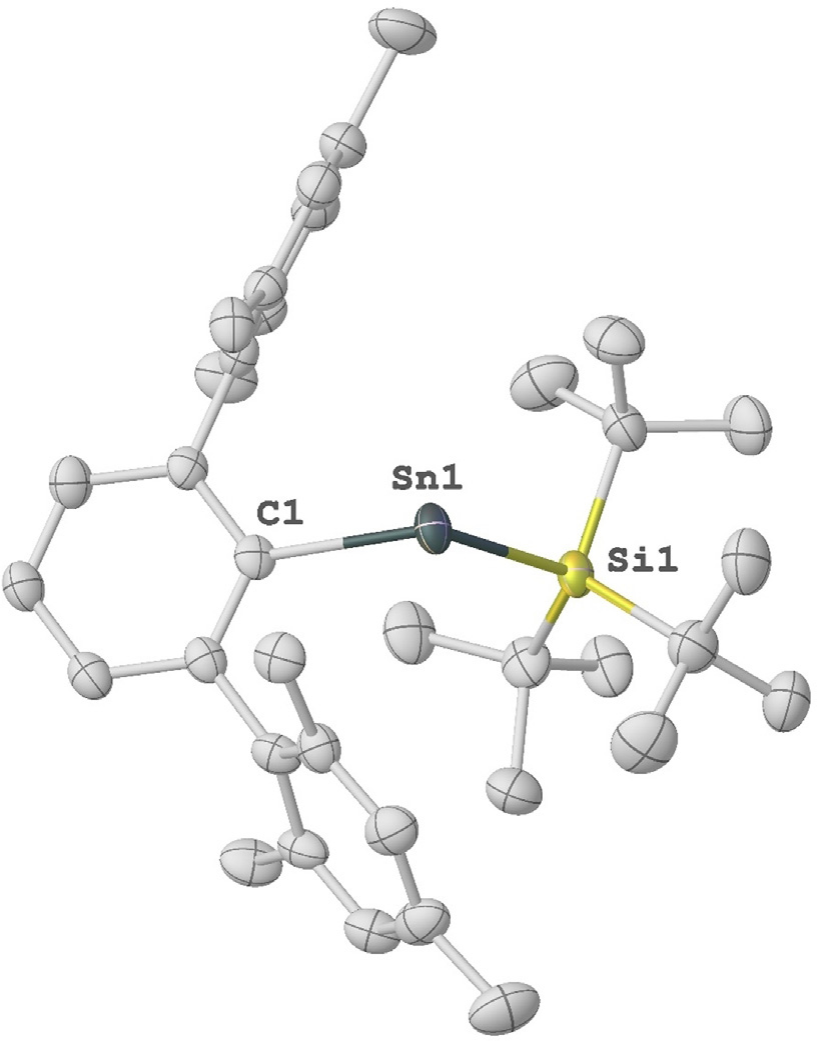
Molecular structure of compound **2** in the solid state. Ellipsoids are set at the 50 % probability level; hydrogen atoms are omitted for clarity. Selected bond lengths [Å] and bond angles [°]: Si1–Sn1 2.6981(17), Sn1–C1 2.217(5), C1‐Sn1‐Si1 113.50(14).

To understand the electronic structure of **2**, we performed a computational analysis. The HOMO (highest occupied molecular orbital) and LUMO (lowest unoccupied molecular orbital) of **2** (Figure 2 A) are centered at the Sn moiety and represent the filled p_*x*_‐ and empty p_*z*_‐orbitals of the tin center. The two frontier orbitals are separated by 3.08 eV (Δ*E* ^s/*t*^=119 kJ mol^−1^; Figure 2 B, left), which is considerably smaller than for the reported diaryl stannylene (^Mes^Ter_2_Sn, 3.41 eV; Δ*E* ^s/*t*^=152 kJ mol^−1^; Figure 2 B, right) and suggests higher reactivity for the former. Most salient, whilst the energy levels of the LUMOs are essentially equivalent (**2**, −1.88 eV; ^Mes^Ter_2_Sn, −1.90 eV), the HOMO of **2**, associated with the p_*x*_ orbital and showing overlap with the Si atom, is much higher in energy (**2**, −4.96 eV; ^Mes^Ter_2_Sn, −5.31 eV). This corroborates that enhanced σ‐donation from the silyl substituent considerably enhances the nucleophilicity of **2** in relation to the bis(aryl)‐substituted congener, whereas the electrophilicity of both compounds should be similar.

**Figure 2:**
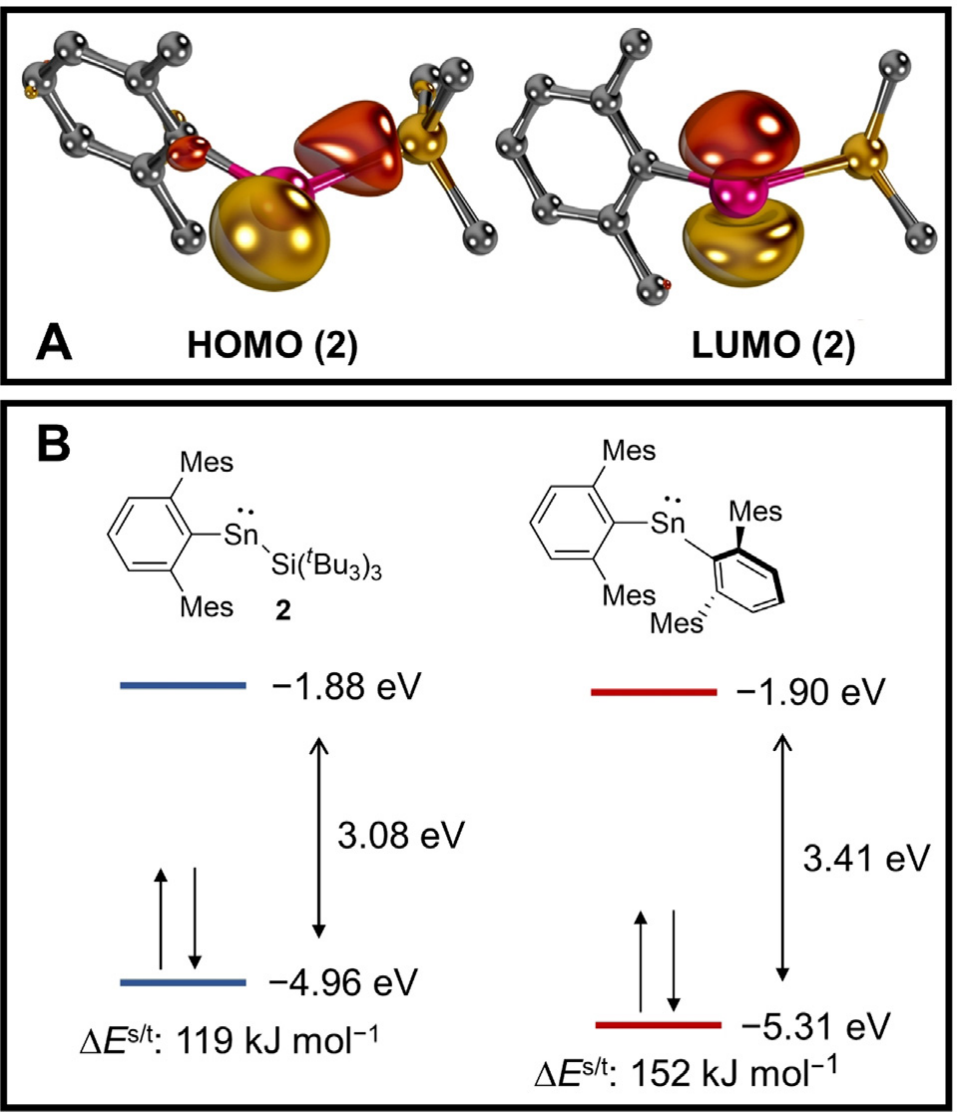
Frontier orbitals of **2** (A), their energies and vertical singlet/triplet gap Δ*E* ^s/t^ (B, left) and comparison with ^Mes^Ter_2_Sn (B, right) as obtained at the PBE0‐D3/def2‐TZVPP//PBE0‐D3/def2‐SVP level of theory.

Encouraged by the computational results, we hypothesized whether compound **2** may activate P_4_. A trial NMR scale reaction of **2** with P_4_ at room temperature afforded an immediate color change from blue to blue‐green and a yellow solution was obtained within 15 min. Multinuclear NMR analysis confirmed the quantitative conversion of **2** to a tin polyphosphide complex. The ^31^P{^1^H} NMR spectrum revealed resonances for three distinct ^31^P nuclei (X, A and B) at *δ*_X_=134.3, *δ*_A_=−211.9 and *δ*_B_=−278.2 ppm. Interestingly, the downfield resonance signal *δ*_X_ is split into a doublet of doublets (^1^ *J*(P_X_,P_A_)=159.0, ^1^ *J*(P_X_,P_B_)=154.8 Hz) while each of the two up‐field signals show a doublet of triplets (^1^ *J*(P_X_,P_A_)=159.0, ^1^ *J*(P_A_,P_B_)=160.7 Hz), indicating an ABX_2_ type splitting pattern. This observation is in line with the isovalent [LSiP_4_, L=β‐diketiminate] complex reported by Driess and co‐workers.^[4a]^ The ^119^Sn spectrum of **3** exhibits a triplet resonance at *δ*=26.3 ppm (^1^ *J*_Sn‐P_=323.8 Hz), which is upfield compared to **1** and falls in the range of known tin polyphosphide complexes (*δ*=+130.4 to −1540.0 ppm).^[13]^ Notably, tin polyphosphide complexes are typically generated by salt metathesis reactions with the metal polyphosphide and stannylene.[^13a^, ^13b^, ^13c^, ^14^] Based on these observations, compound **3** is proposed to contain a coordinated P_4_ unit at the Sn center. Repetition of this reaction on a preparative scale enabled the isolation of compound **3** [^Mes^TerSn(P_4_)Si^*t*^Bu_3_] as a yellow solid in 91 % yield. Thus, facile access to a tin polyphosphide, directly from P_4_, presents an attractive route in P_4_ utilization.

SC‐XRD of **3** confirmed the coordination of ^Mes^TerSn(Si^*t*^Bu_3_) across the P_4_ unit yielding a tetrahedral tin center with a tricyclic SnP_4_ core (Figure 2). Notably, regioselective activation of P_4_ at main group centers is rare.[^1a^, ^1c^] Compound **3** is isostructural to the reported LSiP_4_ and (*m*‐Ter)_2_GeP_4_ complexes (Figure 3).[^4a^, ^4b^] In **3**, two Sn‐P bond lengths are almost identical (Sn1‐P1 2.5714(7) and Sn1‐P4 2.5767(7) Å) and fall within the range of Sn‐P single bonds.[^13b^, ^13c^, ^14^] The exterior P−P bond length of the tetrahedron is P3‐P2 2.1638(10) Å, which is slightly shorter than the P‐P bond length adjacent to the Sn center (P4‐P2 2.2282(9) to P4‐P3 2.2158(10) and P2‐P1 2.2341(10) Å). Interestingly, in compound **3** the C1‐Sn1‐Si1 bond angle (124.77(5)°) is wider than in **2** (113.50(14)°).

**Figure 3:**
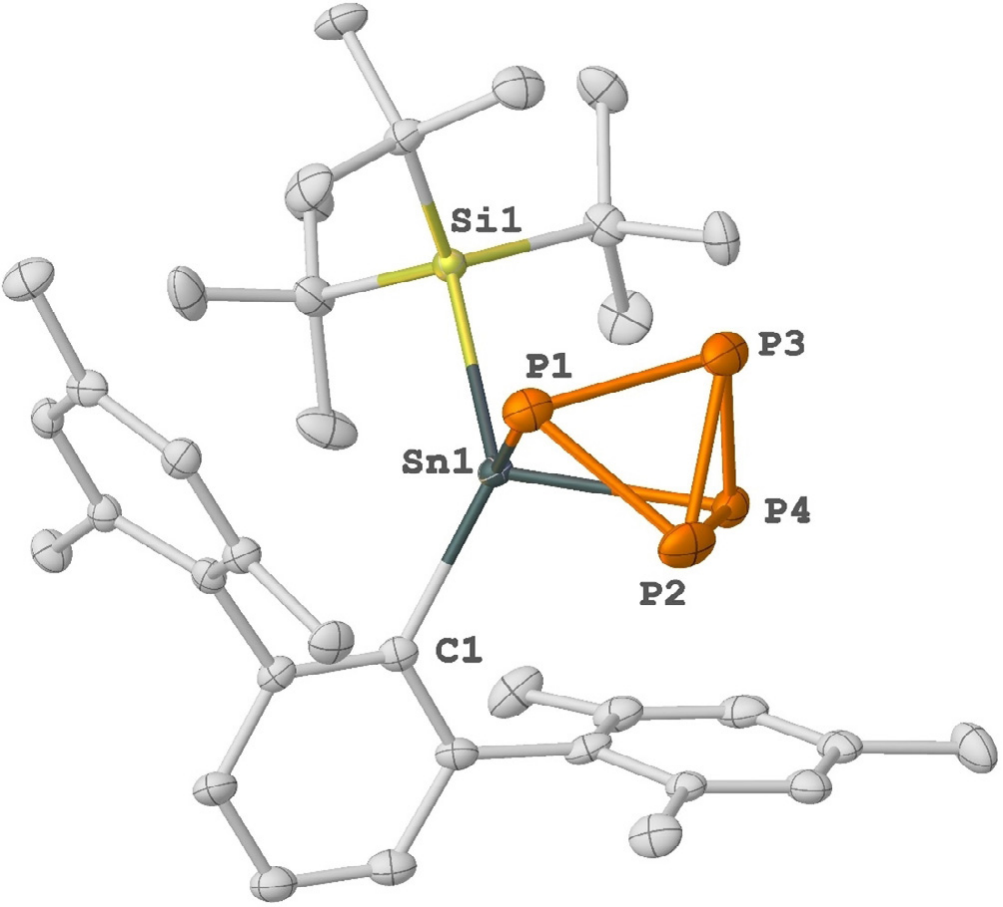
Molecular structures of compound **3** in the solid state. Ellipsoids are set at the 50 % probability level; hydrogen atoms are omitted for clarity. Selected bond lengths [Å] and bond angles [°]: Si1–Sn1 2.6960(7), Sn1–C1 2.223(2), Sn1–P1 2.5714(7), Sn1–P4 2.5767(7), P2–P3 2.1638(10), P4–P3 2.2158(10), P4–P2 2.2282(9), P2–P1 2.2341(10), P3–P1 2.2260(10), C1‐Sn1‐Si1 124.77(5).

Further calculations at the DLPNO‐CCSD(T)/def2‐TZVPP//PBE0‐D3/def2‐SVP level of theory with consideration of solvation effects (SMD) shed further light on both the kinetics as well as thermodynamics of the conversion of **2** to **3** compared to the bis(aryl)stannylene system (Figure 4). The barrier for the former (Δ*G*=+86 kJ mol^−1^) was found to be 19 kJ mol^−1^ lower than for the latter (Δ*G*=+105 kJ mol^−1^). Accordingly, orbital overlap in the asymmetric (Sn‐P1: 2.91 Å; Sn‐P2: 2.78 Å) transition state further corroborates a dominating nucleophilic interaction in the overall ambiphilic activation step. This highlights the influence of the electropositive silyl group on the Sn^II^ center in enabling the activation of P_4_ in contrast to the bis(aryl) system and corroborates our overall design principle.

**Figure 4:**
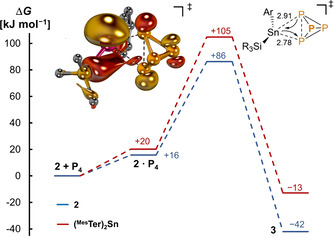
Gibbs free energy profile for P_4_ activation by ^Mes^TerSn(Si^*t*^Bu_3_) **2** [blue —] and (^Mes^Ter)_2_Sn [red —] obtained at the DLPNO‐CCSD(T)/def2‐TZVPP//PBE0‐D3/def2‐SVP level of theory and bond lengths, given in Å, as well as HOMO of transition state. Mesityl and *t*Bu substituents as well as hydrogen atoms are omitted for clarity.

Interestingly, on storage of a [D_8_]THF solution of compound **3** under light for one day, it reverted to the blue‐green color of compound **2**. Multinuclear NMR analysis (^1^H and ^31^P NMR) suggested the presence of both compounds **3** and **2** in solution, as well as free P_4_. The conversion of **3** to **2** is further facilitated by using a UV light source with a range of (300–400 nm) with the liberation of P_4_ observed after 1 h due to the characteristic color change (yellow (**3**) to blue‐green (**2**)) and confirmed by multinuclear NMR (^31^P and ^1^H). Indeed, time‐dependent DFT (TD‐DFT) calculations corroborate that the transitions to the S_2_ state (*f* ^osc^=0.11), experimentally observed at 351 nm (Figure S12), relates to a transition from an essentially Sn‐P_4_ bonding‐ to a Sn‐P_4_ antibonding orbital (Figure S17). Whilst quantitative conversion of **3** to **2** was not achieved, even after prolonged irradiation, this study pointed towards the reversible addition of P_4_ across the Sn^II^ center. The low conversion of **3** to **2** is attributed to the facile activation of P_4_ with **2**, as the equilibrium of this reaction (cf. Figure 4) should be entirely on the product side in contrast to (^Mes^Ter)_2_Sn (Δ*G*=−42 kJ mol^−1^ vs. −13 kJ mol^−1^). Additionally, the barrier for activation of P_4_ is low (Δ*G* ^≠^=+86 kJ mol^−1^), consistent with a fast reaction at room temperature. Notably, compound **2** demonstrates the first example of reversible P−P bond activation with low valent tin compounds.

Encouraged by the reversible activation of P_4_, we were interested to see if **3** could be used as a P_4_ transfer reagent. In a similar fashion to that reported by Scheer and co‐workers, on treatment of **3** with three equivalent of silylene chloride ([PhC(N^*t*^Bu)_2_SiCl]) the yellow solution immediately turned to orange (Scheme 2).^[15]^ The ^31^P NMR spectrum revealed a mixture of phosphorus containing compounds, however, distinct resonance signals were observed for compound **4** [{PhC(N^*t*^Bu)_2_}SiP]_3_ (crude yield=10 %) and **5** [{PhC(N^*t*^Bu)_2_}SiCl}P{SiCl_2_{PhC(N^*t*^Bu)_2_}], at −244.1 and −183.5 ppm, respectively (crude yield=45 %).[^15^, ^16^]

**Scheme 2:**
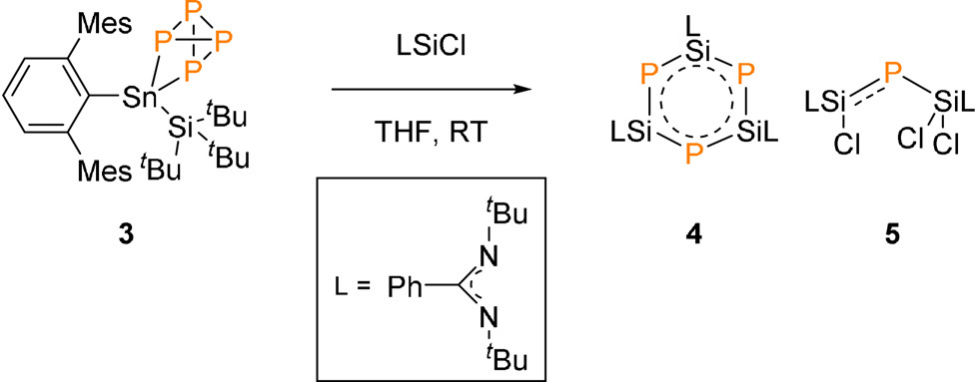
P_4_ transfer reaction.

In summary, we have reported for the first time the reversible P‐P bond activation by a heteroleptic stannylene **2**. Both experimental and computational investigations revealed the effectiveness of the silyl ligand in order to tune and enhance the reactivity of low valent Sn^II^ center. Additionally, transfer of P_4_ to organic molecules has been demonstrated and further functionalization reactivity is currently under investigation in our lab.

## Conflict of interest

The authors declare no conflict of interest.

## Supporting information

As a service to our authors and readers, this journal provides supporting information supplied by the authors. Such materials are peer reviewed and may be re‐organized for online delivery, but are not copy‐edited or typeset. Technical support issues arising from supporting information (other than missing files) should be addressed to the authors.

Supplementary

Supplementary
