# Supplementary material for: Reversible Activation and Transfer of White Phosphorus by Silyl‐Stannylene
Source: Angew Chem Int Ed Engl. 2020 Dec 21;60(7):3519–23. doi: 10.1002/anie.202013423 (PMC7898380; doi:10.1002/anie.202013423)

checkCIF/PLATON report

Structure factors have been supplied for datablock(s) cu-3601_tw

THIS REPORT IS FOR GUIDANCE ONLY. IF USED AS PART OF A REVIEW PROCEDURE FOR PUBLICATION, IT SHOULD NOT REPLACE THE EXPERTISE OF AN EXPERIENCED CRYSTALLOGRAPHIC REFEREE.

No syntax errors found.   CIF dictionary   Interpreting this report

Datablock: cu-3601_tw

```
Bond precision:  C-C = 0.0092 A          Wavelength=1.54184

Cell:            a=11.1308(3)     b=18.2702(4)      c=16.1897(4)
                 alpha=90         beta=91.298(2)    gamma=90
Temperature:     150 K

                Calculated                 Reported
Volume          3291.52(14)                3291.52(14)
Space group     P 21/n                     P 1 21/n 1
Hall group      -P 2yn                     -P 2yn
Moiety formula  C36 H52 Si Sn              C36 H52 Si Sn
Sum formula     C36 H52 Si Sn              C36 H52 Si Sn
Mr              631.58                     631.55
Dx,g cm-3       1.275                      1.274
Z               4                          4
Mu (mm-1)       6.664                      6.664
F000            1328.0                     1328.0
F000'           1331.80
h,k,lmax        13,22,20                   13,22,19
Nref            6666                       6514
Tmin,Tmax       0.347,0.587                0.667,0.778
Tmin'           0.262

Correction method= # Reported T Limits: Tmin=0.667 Tmax=0.778
AbsCorr = MULTI-SCAN

Data completeness= 0.977          Theta(max)= 73.837

R(reflections)= 0.0808( 5454)     wR2(reflections)= 0.2493( 6514)

S = 1.070                  Npar= 359
```

The following ALERTS were generated. Each ALERT has the format
test-name_ALERT_alert-type_alert-level.
Click on the hyperlinks for more details of the test.

Alert level C

```
PLAT342_ALERT_3_C Low Bond Precision on  C-C Bonds ...............    0.00917 Ang.
PLAT911_ALERT_3_C Missing FCF Refl Between Thmin & STh/L=    0.600         23 Report
```

Alert level G

```
PLAT072_ALERT_2_G SHELXL First  Parameter in WGHT  Unusually Large       0.19 Report
PLAT720_ALERT_4_G Number of Unusual/Non-Standard Labels ..........          4 Note
PLAT870_ALERT_4_G ALERTS Related to Twinning Effects Suppressed ..          ! Info
PLAT883_ALERT_1_G No Info/Value for _atom_sites_solution_primary .     Please Do !
PLAT912_ALERT_4_G Missing # of FCF Reflections Above STh/L=  0.600        121 Note
PLAT931_ALERT_5_G CIFcalcFCF  Twin Law ( 1 0 0)         Est.d BASF       0.40 Check
PLAT931_ALERT_5_G CIFcalcFCF  Twin Law ( 0 0 1)         Est.d BASF       0.34 Check
PLAT933_ALERT_2_G Number of OMIT Records in Embedded .res File ...         23 Note
PLAT941_ALERT_3_G Average HKL Measurement Multiplicity ...........        1.0 Low
```

0 **ALERT level A** = Most likely a serious problem - resolve or explain
0 **ALERT level B** = A potentially serious problem, consider carefully
2 **ALERT level C** = Check. Ensure it is not caused by an omission or oversight
9 **ALERT level G** = General information/check it is not something unexpected

1 ALERT type 1 CIF construction/syntax error, inconsistent or missing data
2 ALERT type 2 Indicator that the structure model may be wrong or deficient
3 ALERT type 3 Indicator that the structure quality may be low
3 ALERT type 4 Improvement, methodology, query or suggestion
2 ALERT type 5 Informative message, check

It is advisable to attempt to resolve as many as possible of the alerts in all categories. Often the minor alerts point to easily fixed oversights, errors and omissions in your CIF or refinement strategy, so attention to these fine details can be worthwhile. In order to resolve some of the more serious problems it may be necessary to carry out additional measurements or structure refinements. However, the purpose of your study may justify the reported deviations and the more serious of these should normally be commented upon in the discussion or experimental section of a paper or in the "special_details" fields of the CIF. checkCIF was carefully designed to identify outliers and unusual parameters, but every test has its limitations and alerts that are not important in a particular case may appear. Conversely, the absence of alerts does not guarantee there are no aspects of the results needing attention. It is up to the individual to critically assess their own results and, if necessary, seek expert advice.

Publication of your CIF in IUCr journals

A basic structural check has been run on your CIF. These basic checks will be run on all CIFs submitted for publication in IUCr journals (*Acta Crystallographica*, *Journal of Applied Crystallography*, *Journal of Synchrotron Radiation*); however, if you intend to submit to *Acta Crystallographica Section C* or *E* or *IUCrData*, you should make sure that full publication checks are run on the final version of your CIF prior to submission.

Publication of your CIF in other journals

Please refer to the *Notes for Authors* of the relevant journal for any special instructions relating to CIF submission.

PLATON version of 18/09/2020; check.def file version of 20/08/2020

Datablock cu-3601_tw - ellipsoid plot

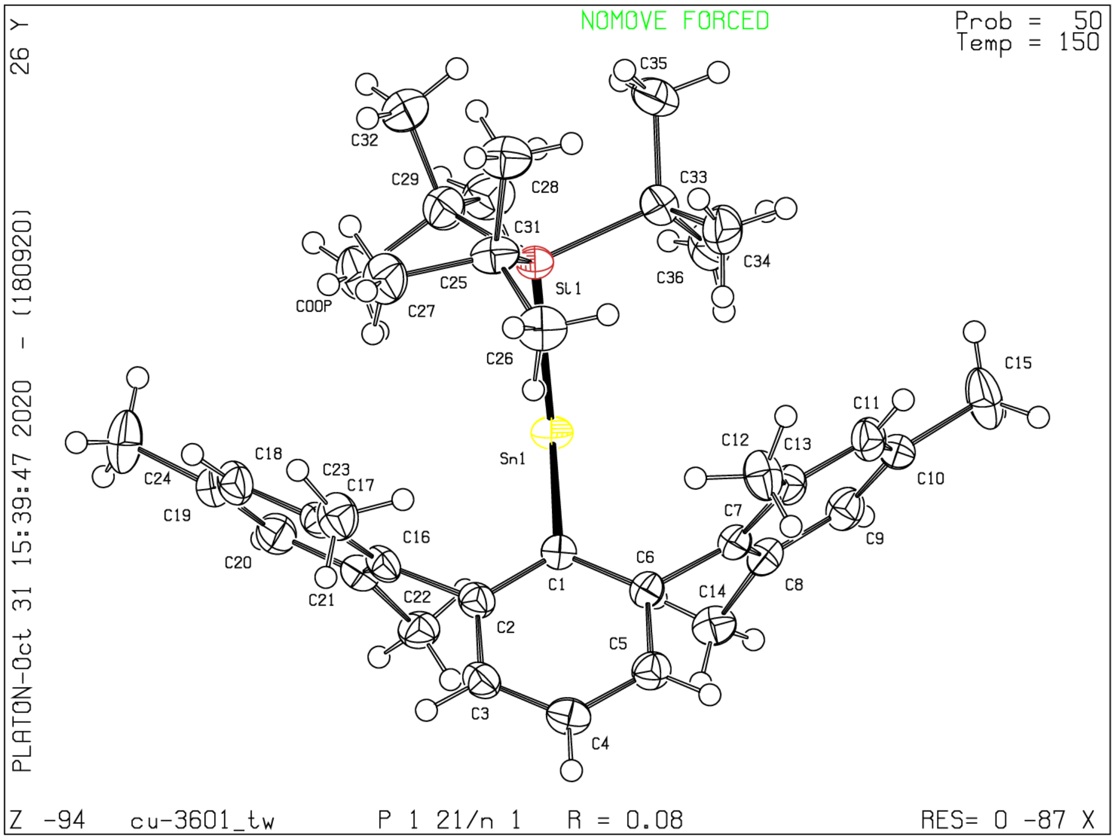

Supplement: Supplementary file 1 — Supplementary [file ANIE-60-3519-s001.zip › compound_2_checkcif.pdf]
