# Supplementary material for: Reversible Activation and Transfer of White Phosphorus by Silyl‐Stannylene
Source: Angew Chem Int Ed Engl. 2020 Dec 21;60(7):3519–23. doi: 10.1002/anie.202013423 (PMC7898380; doi:10.1002/anie.202013423)
Supplement: Supplementary file 1 — Supplementary [file ANIE-60-3519-s001.zip › compund_3_checkcif.pdf]

checkCIF () running

Checking for embedded fcf data in CIF ...
Found embedded fcf data in CIF. Extracting fcf data from uploaded CIF, please wait ....

checkCIF/PLATON (full publication check)

You have not supplied any structure factors. As a result the full set of tests cannot be run.

THIS REPORT IS FOR GUIDANCE ONLY. IF USED AS PART OF A REVIEW PROCEDURE FOR PUBLICATION, IT SHOULD NOT REPLACE THE EXPERTISE OF AN EXPERIENCED CRYSTALLOGRAPHIC REFEREE.You have not supplied any structure factors. As a result the full set of tests cannot be run.

No syntax errors found.
Please wait while processing ....
Structure factor report

CIF dictionary
Interpreting this report

Datablock: I

Bond precision: C-C = 0.0033 A Wavelength=0.71073

Cell:	a=19.2663(11)	b=11.4979(8)	c=16.5652(11)
	alpha=90	beta=90	gamma=90
Temperature:	100 K		

	Calculated	Reported
Volume	3669.6(4)	3669.6(4)
Space group	P n a 21	P n a 21
Hall group	P 2c -2n	P 2c -2n
Moiety formula	C36 H52 P4 Si Sn	C36 H52 P4 Si Sn
Sum formula	C36 H52 P4 Si Sn	C36 H51 P4 Si Sn
Mr	755.46	754.42
Dx,g cm-3	1.367	1.366
Z	4	4
Mu (mm-1)	0.927	0.927
F000	1568.0	1564.0
F000'	1567.68	
h,k,lmax	23,14,20	23,14,20
Nref	6980[3619]	6967
Tmin,Tmax	0.816,0.911	0.720,0.745
Tmin'	0.816	

Correction method= # Reported T Limits: Tmin=0.720 Tmax=0.745 AbsCorr = MULTI-SCAN

Data completeness= 1.93/1.00 Theta(max)= 25.703

R(reflections)= 0.0133(6885) wR2(reflections)= 0.0348(6967)

S = 1.037 Npar= 395

The following ALERTS were generated. Each ALERT has the format
test-name_ALERT_alert-type_alert-level.
Click on the hyperlinks for more details of the test.

Alert level B

PLAT107_ALERT_2_B Twinning Matrix Invalid in Centrosymmetric SPGR ? Check

Alert level C

STRVA01_ALERT_4_C Flack test results are ambiguous.
From the CIF: _refine_ls_abs_structure_Flack 0.437
From the CIF: _refine_ls_abs_structure_Flack_su 0.011
PLAT041_ALERT_1_C Calc. and Reported SumFormula Strings Differ Please Check
PLAT043_ALERT_1_C Calculated and Reported Mol. Weight Differ by .. 1.04 Check
PLAT068_ALERT_1_C Reported F000 Differs from Calcd (or Missing)... Please Check
PLAT761_ALERT_1_C CIF Contains no X-H Bonds .... Please Check
PLAT762_ALERT_1_C CIF Contains no X-Y-H or H-Y-H Angles .... Please Check

Alert level G

FORMU01_ALERT_1_G There is a discrepancy between the atom counts in the
_chemical_formula_sum and _chemical_formula_moiety. This is
usually due to the moiety formula being in the wrong format.
Atom count from _chemical_formula_sum: C36 H51 P4 Si1 Sn1
Atom count from _chemical_formula_moiety:C36 H52 P4 Si1 Sn1
FORMU01_ALERT_2_G There is a discrepancy between the atom counts in the
_chemical_formula_sum and the formula from the _atom_site* data.
Atom count from _chemical_formula_sum:C36 H51 P4 Si1 Sn1
Atom count from the _atom_site data: C36 H52 P4 Si1 Sn1
CELLZ01_ALERT_1_G Difference between formula and atom_site contents detected.
CELLZ01_ALERT_1_G ALERT: Large difference may be due to a
symmetry error - see SYMMG tests
From the CIF: _cell_formula_units_Z 4
From the CIF: _chemical_formula_sum C36 H51 P4 Si Sn
TEST: Compare cell contents of formula and atom_site data

atom	Z*formula	cif sites	diff
C	144.00	144.00	0.00
H	204.00	208.00	-4.00
P	16.00	16.00	0.00
Si	4.00	4.00	0.00
Sn	4.00	4.00	0.00

PLAT232_ALERT_2_G Hirshfeld Test Diff (M-X) Sn1 --P1 . 7.3 s.u.
PLAT232_ALERT_2_G Hirshfeld Test Diff (M-X) Sn1 --P4 . 7.3 s.u.
PLAT328_ALERT_4_G Possible Missing H on sp3? Phosphorus .... P1 Check
PLAT328_ALERT_4_G Possible Missing H on sp3? Phosphorus .... P4 Check
PLAT870_ALERT_4_G ALERTS Related to Twinning Effects Suppressed .. ! Info

0 **ALERT level A** = Most likely a serious problem - resolve or explain
1 **ALERT level B** = A potentially serious problem, consider carefully
6 **ALERT level C** = Check. Ensure it is not caused by an omission or oversight
9 **ALERT level G** = General information/check it is not something unexpected

8 ALERT type 1 CIF construction/syntax error, inconsistent or missing data
4 ALERT type 2 Indicator that the structure model may be wrong or deficient
0 ALERT type 3 Indicator that the structure quality may be low
4 ALERT type 4 Improvement, methodology, query or suggestion
0 ALERT type 5 Informative message, check

checkCIF publication errors

Alert level A

PUBL006_ALERT_1_A _publ_requested_journal is missing
e.g. 'Acta Crystallographica Section C'

1 **ALERT level A** = Data missing that is essential or data in wrong format
0 **ALERT level G** = General alerts. Data that may be required is missing

Publication of your CIF
You should attempt to resolve as many as possible of the alerts in all categories. Often the minor alerts point to easily fixed

oversights, errors and omissions in your CIF or refinement strategy, so attention to these fine details can be worthwhile. In order to resolve some of the more serious problems it may be necessary to carry out additional measurements or structure refinements. However, the nature of your study may justify the reported deviations from journal submission requirements and the more serious of these should be commented upon in the discussion or experimental section of a paper or in the "special_details" fields of the CIF. *checkCIF* was carefully designed to identify outliers and unusual parameters, but every test has its limitations and alerts that are not important in a particular case may appear. Conversely, the absence of alerts does not guarantee there are no aspects of the results needing attention. It is up to the individual to critically assess their own results and, if necessary, seek expert advice.
If level A alerts remain, which you believe to be justified deviations, and you intend to submit this CIF for publication in a journal, you should additionally insert an explanation in your CIF using the Validation Reply Form (VRF) below. This will allow your explanation to be considered as part of the review process.

Validation response form

Please find below a validation response form (VRF) that can be filled in and pasted into your CIF.

```
# start Validation Reply Form
_vrf_PUBL006_GLOBAL
;
PROBLEM: _publ_requested_journal is missing
RESPONSE: ...
;
# end Validation Reply Form
```

If you wish to submit your CIF for publication in Acta Crystallographica Section C or E, you should upload your CIF via the web. If you wish to submit your CIF for publication in IUCrData you should upload your CIF via the web. If your CIF is to form part of a submission to another IUCr journal, you will be asked, either during electronic submission or by the Co-editor handling your paper, to upload your CIF via our web site.

PLATON version of 18/09/2020; check.def file version of 20/08/2020

Datablock I - ellipsoid plot

Download CIF editor (publCIF) from the IUCr
Download CIF editor (enCIFer) from the CCDC
Test a new CIF entry
