# Supplementary material for: Reversible Activation and Transfer of White Phosphorus by Silyl‐Stannylene
Source: Angew Chem Int Ed Engl. 2020 Dec 21;60(7):3519–23. doi: 10.1002/anie.202013423 (PMC7898380; doi:10.1002/anie.202013423)
Supplement: Supplementary file 2 — Supplementary [file ANIE-60-3519-s002.pdf]

Angewandte Chemie
Eine Zeitschrift der Gesellschaft Deutscher Chemiker

Supporting Information

Reversible Activation and Transfer of White Phosphorus by Silyl-Stannylene

*Debotra Sarkar, Catherine Weetman, Dominik Munz, and Shigeyoshi Inoue**

anie_202013423_sm_miscellaneous_information.pdf
anie_202013423_sm_cif.zip

Supporting Information

Contents

1. Experimental Details 1
1.1 General Information 1
1.2 Synthesis of compound 2: 2
1.3 Synthesis of compound 3: 4
1.4 Preparation of compounds 4 and 5: 10
2. X-Ray Crystallography 11
3. Computations 13
3.1 General 13
3.2 Energies 14
3.3 XYZ Coordinates 15
4. References 22

1. Experimental Details

1.1 General Information

All experiments and manipulations were carried out under a dry argon atmosphere using standard Schlenk techniques or in a glovebox. All glass junctions were coated with PTFE-based grease Merkel Triboflon III. n-Hexane, n-Pentane, THF, Benzene and toluene were refluxed over sodium/benzophenone, freshly distilled and deoxygenated prior to use. The ^{1}H, ^{13}C, ^{29}Si and ^{31}P NMR spectra were measured Bruker 400 MHz and 500 MHz spectrometers. Chemical shifts were referenced to residual solvent signals (^{1}H and ^{13}C NMR). ^{119}Sn NMR chemical shifts was referenced to Me_4Sn (^{119}Sn). Deuterated solvent C_6D_6 and THF-D_8 were obtained from Deutero Deutschland GmbH and were dried over 4 Å molecular sieves prior to use. White phosphorus (P_4) was sublimed and stored at –35 °C in the dark in the glovebox. Unless otherwise stated, all reagents were purchased from commercial sources and used as received. Abbreviations: s = singlet, br = broad, d = doublet, t = triplet, m = multiplet. Elemental analyses (EA) were conducted with a EURO EA (HEKA tech) instrument equipped with a CHNS combustion analyzer. Analysis of molecular masses were performed on a Thermo Fisher Scientific Exactive Plus Orbitrap system (ionization method: LIFDI). Thereby, all samples were prepared in THF solutions, filtered and injected into the spectrometers. TOF analyzation in cationic mode resulted the obtained spectra, which were resolved by mass-to-charge values. IMe_4 and [^{m}TerSnCl] complex **1** were synthesized according to literature procedures.[1]

1.2 Synthesis of compound 2:

A benzene (2 mL) solution of NaSi*t*-$Bu_3(THF)_2$ (0.39 g, 1.07 mmol, 1.00 eq) was added to ^{Mes}TerSnCl (0.5 g, 1.07 mmol, 1.00 eq) in benzene (2 mL) at room temperature. The color of the solution rapidly changed from orange to blue with formation of a blue precipitate. After stirring the solution for 3 h, the solvent was removed in vacuo. The obtained residue was washed with hexane (3 × 5 mL), extracted with a mixture of benzene (10 mL) + THF (15 mL) and filtered through a microfiber glass filter. The solution was concentrated to approximately 3 mL and pentane (5 mL) was added to aid crystallization. The solution was placed at −25°C and after six days compound **2** was obtained as an analytically pure blue crystalline material. Yield: 0.54 mg, 0.54 mmol, 80 %.

^{1}H NMR (400.13 MHz, 298 K, THF-D8): δ = 0.93(s, 27H, Si*t*-Bu_3), 2.21(s, 12H, 4x$C^{2,6}$-CH_3, Mes), 2.46 (s, 6H, 2xC^4-CH_3, Mes), 5.55 (d, $^2J_{H,P}$ = 5.0 Hz, $^1J_{H,Si}$ = 237.9 Hz, Si-*H*), 6.76–7.14 (m, 6 H, $C^{3,5}$-H_2, C_6H_3, 2x$C^{3,5}$-*H*, Mes), 7.45 (t, $^3J_{H-H}$ = 7.6 Hz, 1H, C^4-*H*, C_6H_3) ppm. **^{13}C{^{1}H} NMR** (125.83 MHz, 298 K, THF-D8): δ = 20.04-21.37 ($C^{2,4,6}$-CH_3, Mes), 29.07 (C(CH_3)$_3$), 31.81 (*C*(CH_3)$_3$), 127.35-128.99 (2xC^4-Mes), 129.21 (2x$C^{3,5}$-H, Mes), 129.90 (s, $C^{3,5}$-H, C_6H_3), 133.85 (s, C^4-H, C_6H_3), 135.96 (2x$C^{2,6}$-Mes), 136.58 (Sn-*C*, C_6H_3), 137.02 (s, 2xC^1-Mes), 145.33 (s, 2x$C^{2,6}$-C_6H_3) ppm. **^{29}Si{^{1}H} NMR** (99.41 MHz, 298 K, THF-D8): δ = 94.69 ppm. **^{119}Sn{^{1}H} NMR** (149.20 MHz, 298 K, THF-D8): 197.3 ppm. **Anal. Calcd. [%] for $C_{36}H_{52}SiSn$**: C, 68.46; H, 8.30. Found C, 68.16; H,8.09. **LIFDI-MS:** calculated for **[$C_{36}H_{52}SiSn$] :** 632.28602, Found 632.284066.

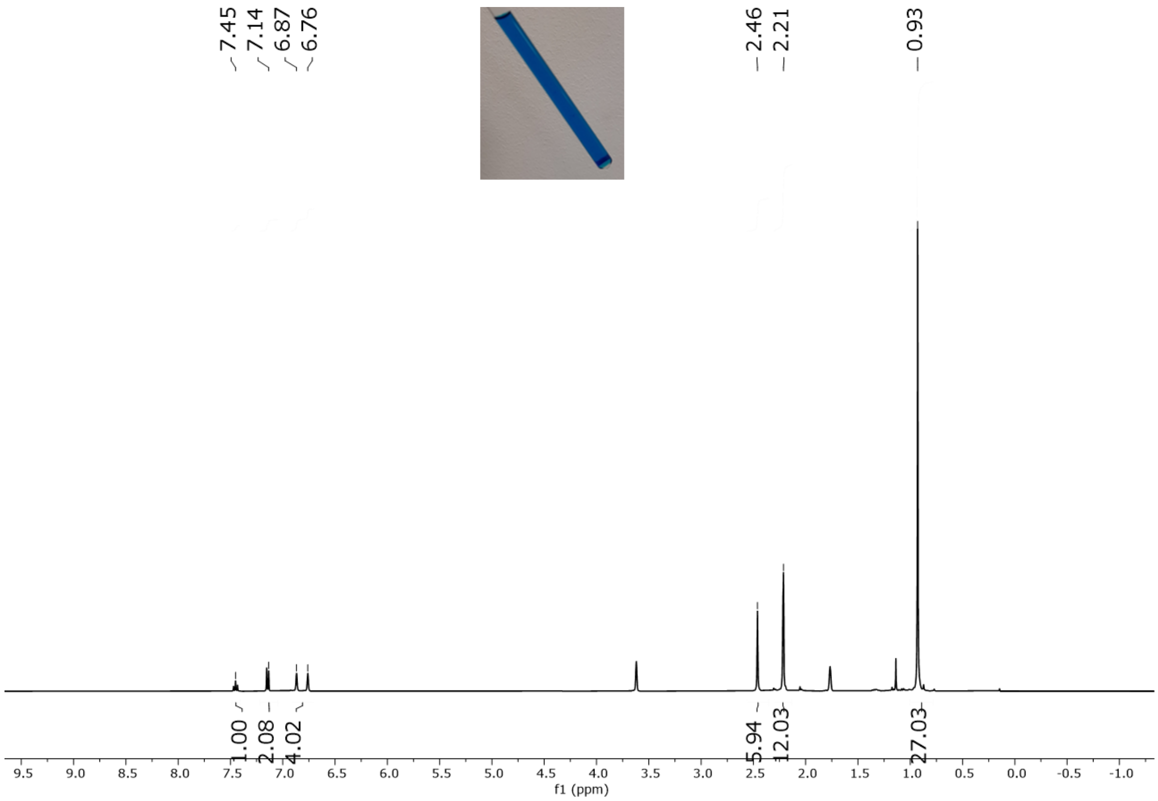

Figure S1. ^{1}H NMR spectrum of compound **2** in THF-D8 at 298 K.

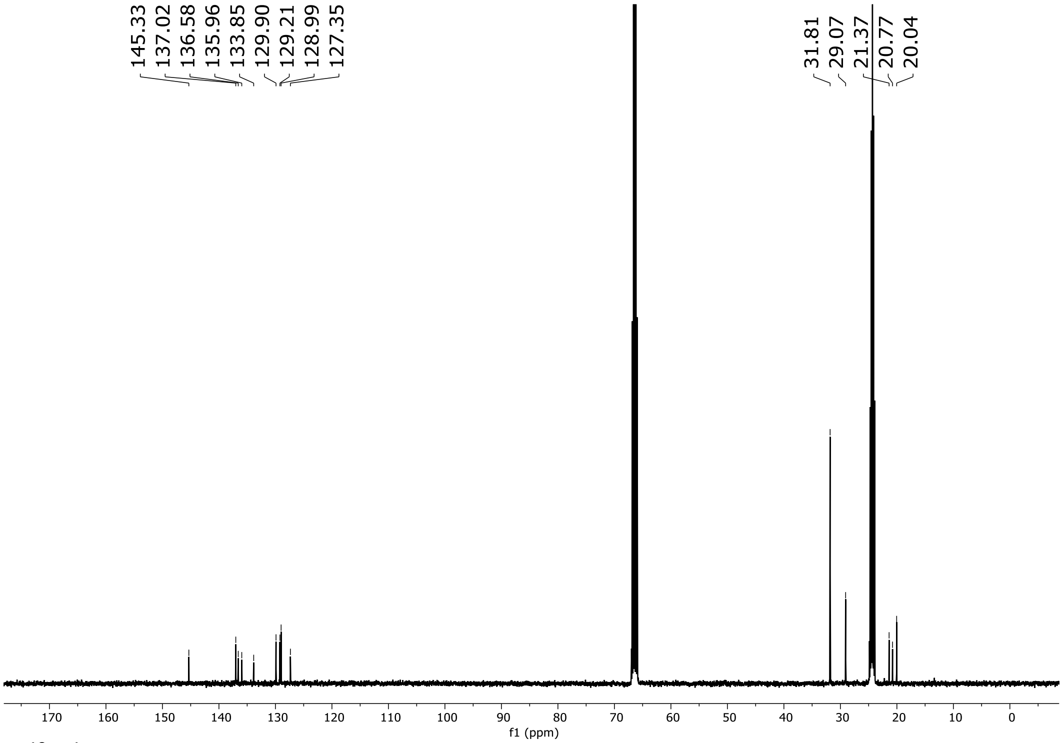

Figure S2. $^{13}C\{^1H\}$ NMR spectrum of compound **2** in THF-D8 at 298 K.

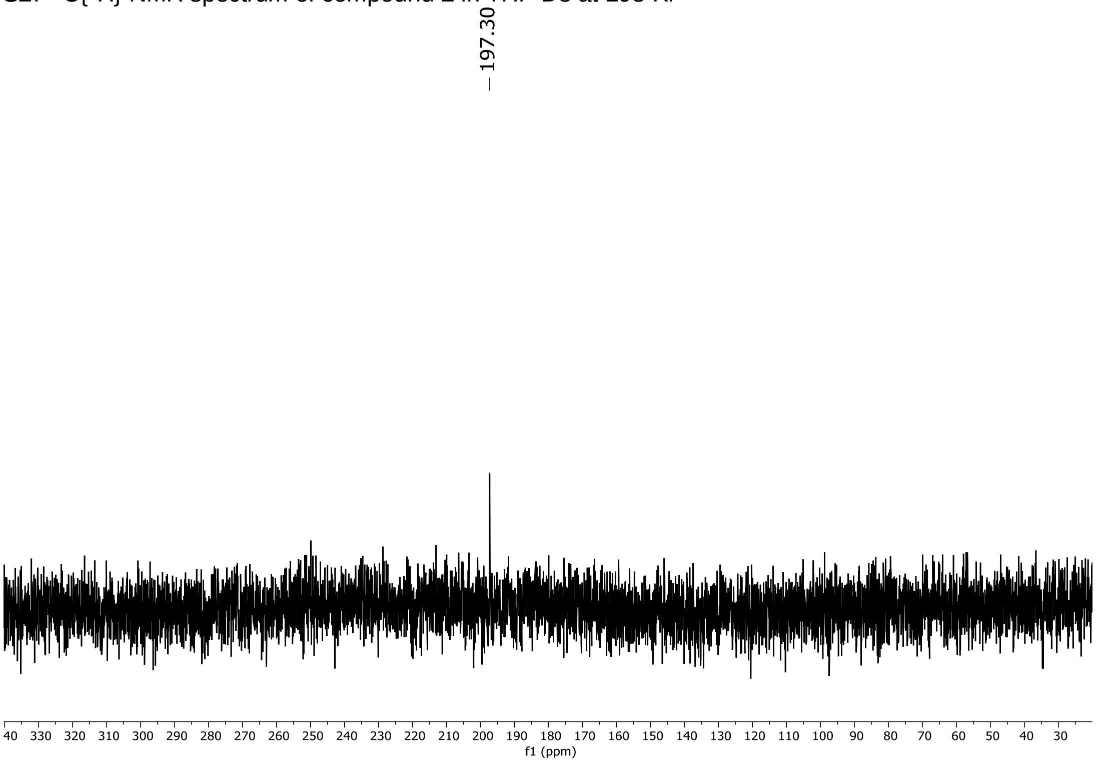

Figure S3. $^{119}Sn\{^1H\}$ NMR spectrum of compound **2** in THF-D8 at 298 K.

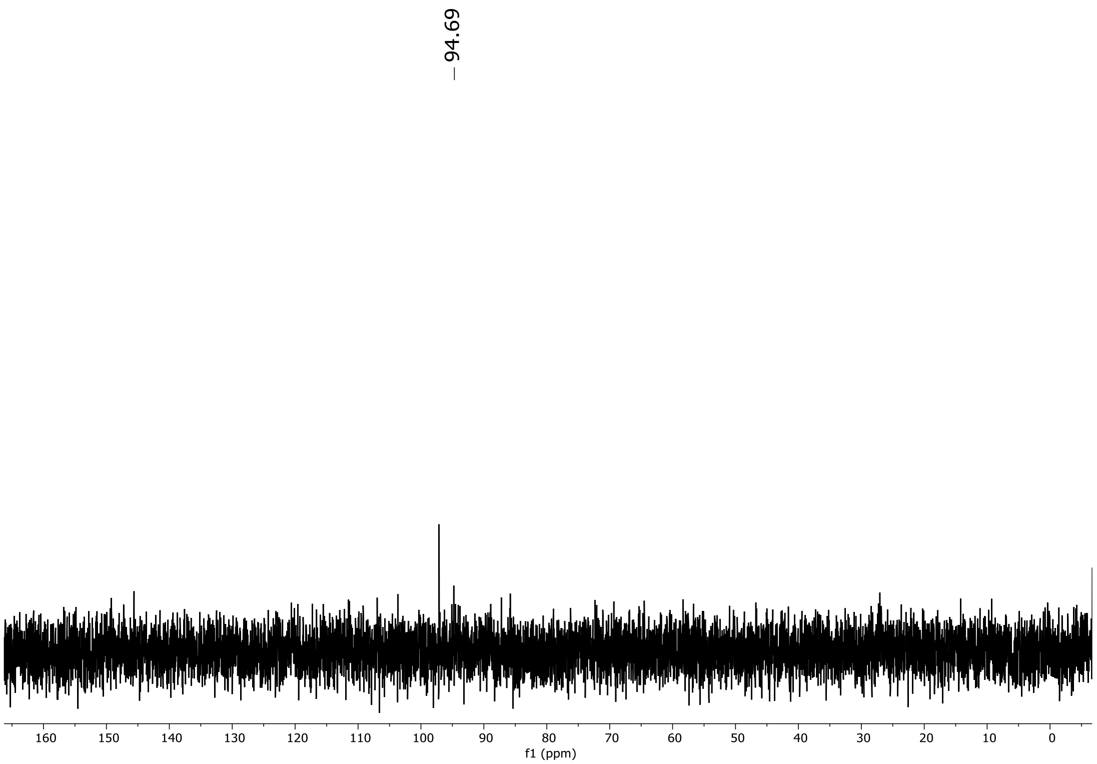

Figure S4. $^{29}Si\{^1H\}$ NMR spectrum of compound **2** in THF-D8 at 298 K.

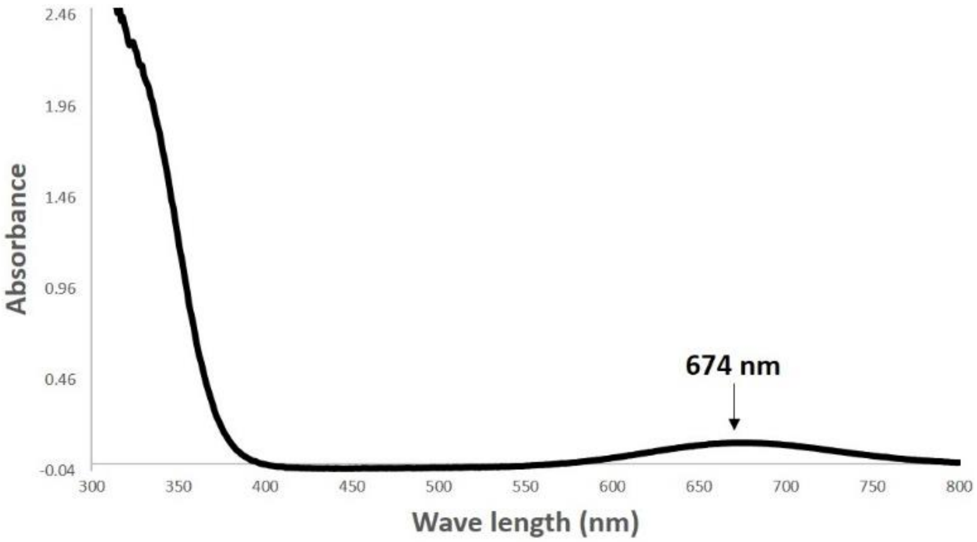

Figure S5. UV-VIS spectrum of compound **2** (THF, 2.5x10^{-3} M) at 298 K.

1.3 Synthesis of compound 3:

A THF (2 mL) solution of **2** (100 mg, 0.16 mmol, 1.00 eq) was added dropwise to a suspension of elemental P_4 (19.6 mg, 0.16 mmol, 1.00 eq) in THF. The blue solution immediately turned to bluish-green and then yellow after 15 min. The solution was stirred for an additional 2 h, followed by the removal of all volatiles. The resulting solid was dissolved in a mixture of Et_2O (2 mL) and n-hexane (2 mL) and placed at -35°C for two days, which yielded yellow crystals of compound **3** (110 mg, 0.15 mmol, 91 %).

^{1}H NMR (400.13 MHz, 298 K, THF-D8): δ = 0.1.28(s, 27H, Si*t*-Bu$_3$), 2.07-2.14(s, 12H, 4xC^{2,6}-C*H*$_3$, Mes), 2.76 (s, 6H, 2xC^{4}-C*H*$_3$, Mes), 6.77–7.16 (m, 6 H, C^{3,5}-*H*$_2$, C$_6$H$_3$, 2xC^{3,5}-*H*, Mes), 7.59 (t, $^{3}J_{H\text{–}H}$ = 7.6 Hz, 1H, C^{4}-*H*, C$_6$H$_3$) ppm. **^{13}C{^{1}H} NMR** (125.83 MHz, 298 K, THF-D8): δ = 19.95-22.23 (C^{2,4,6}-*C*H$_3$, Mes), 27.03 (C(*C*H$_3$)$_3$), 32.12 (*C*(CH$_3$)$_3$), 128.51-129.04 (2x*C*^{4}-Mes), 129.56(2x*C*^{3,5}-H, Mes), 129.84 (s, *C*^{3,5}-H, C$_6$H$_3$), 134.85 (s, *C*^{4}-H, C$_6$H$_3$), 136.43 (2x*C*^{2,6}-Mes), 137.27 (Sn-*C*, C$_6$H$_3$), 139.48(s, 2x*C*^{1}-Mes), 149.24 (s, 2x*C*^{2,6}-C$_6$H$_3$) ppm. **^{29}Si{^{1}H} NMR** (99.41 MHz, 298 K, THF-D8): δ = 53.20 ppm. **^{119}Sn{^{1}H} NMR** (149.20 MHz, 298 K, THF-D8): 26.37 ppm (m,$^{1}J_{Sn,\,P}$ = 323.81 Hz). **^{31}P{^{1}H} NMR** (161.97 MHz, 298 K, THF-D8): 134.31(m,2P,P$_X$, ^{1}J(P$_X$-P$_A$)= 159.08Hz, ^{1}J(P$_X$-P$_B$)= 154.83 Hz), -211.90 (m, P$_A$, ^{1}J(P$_A$-P$_X$) = 159.08Hz, ^{1}J(P$_A$-P$_B$) = 160.71Hz), -278.26 (m, P$_B$, ^{1}J(P$_B$-P$_A$) = 160.71Hz, ^{1}J(P$_B$-P$_X$) =154.83 Hz) ppm. **Anal. Calcd. [%] for C$_{36}$H$_{52}$P$_4$SiSn**: C, 57.23; H, 6.94. Found C, 57.01; H, 6.58. **LIFDI-MS:** calculated for **[C$_{36}$H$_{52}$P$_4$SiSn] :** 756.18107, Found [**(C$_{36}$H$_{52}$P$_4$SiSn)- P4]** 632.28450, **P$_4$** 123.89389.

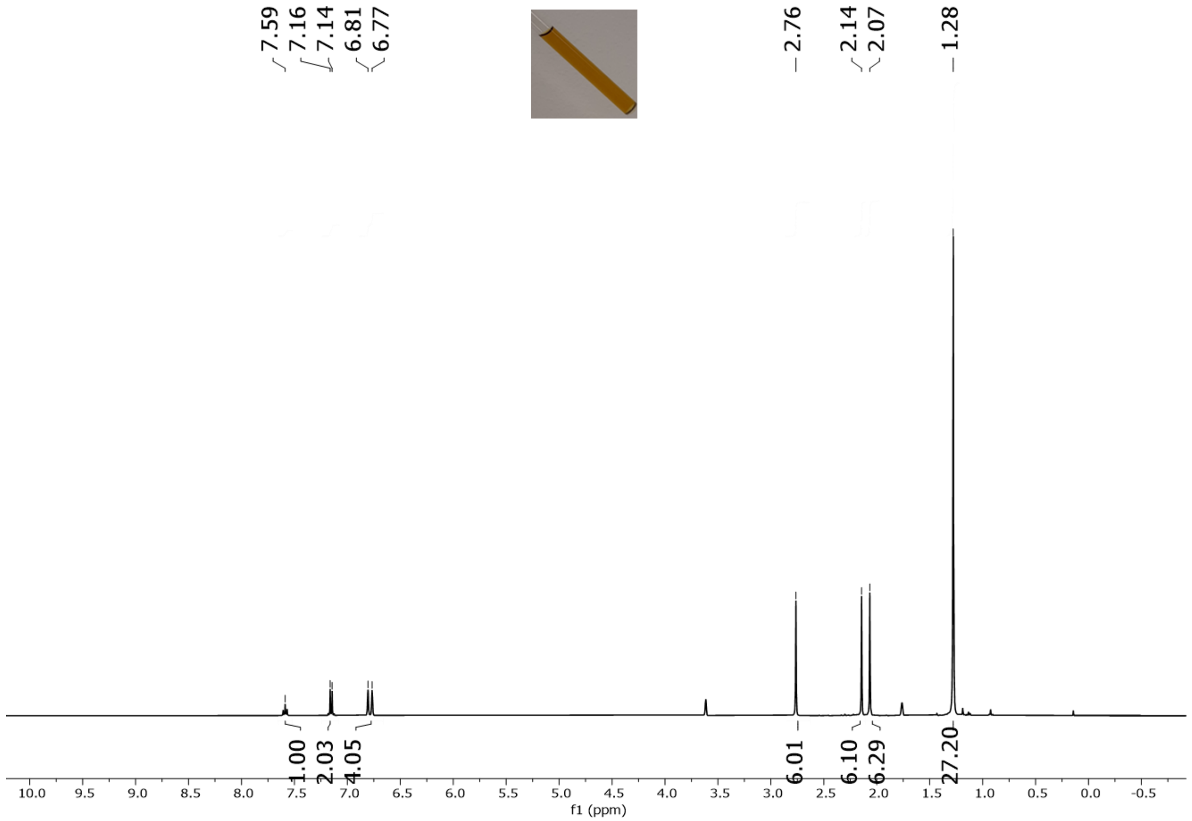

Figure S6. ^{1}H NMR spectrum of compound **3** in THF-D8 at 298 K.

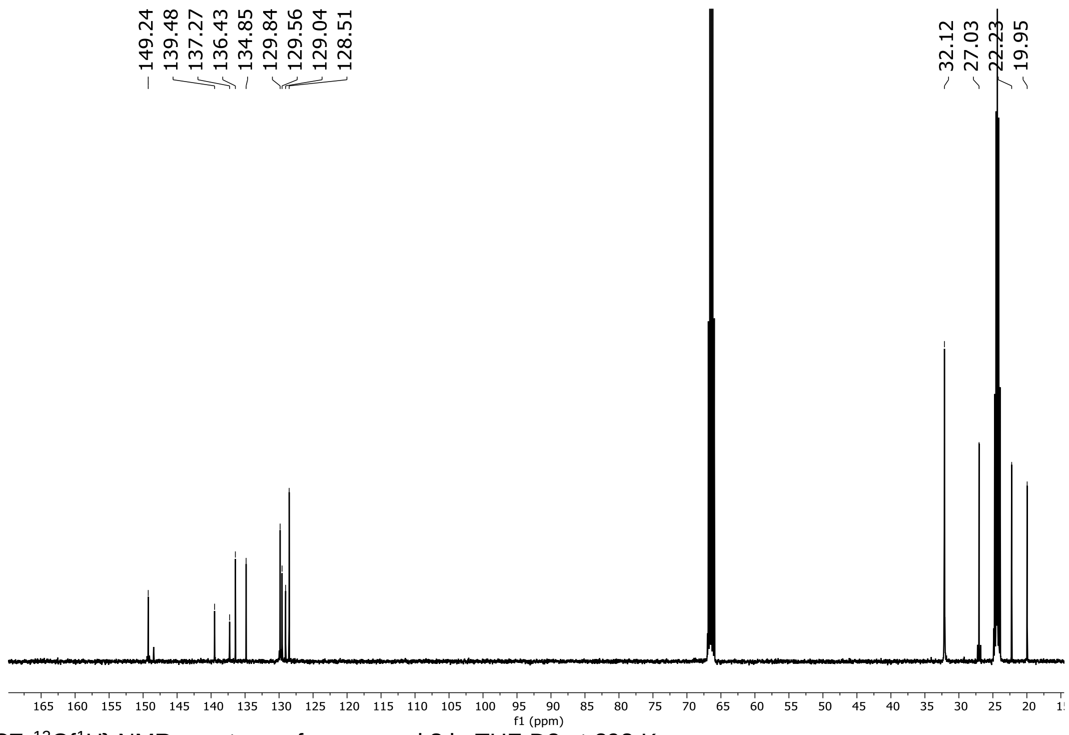

Figure S7. $^{13}C\{^{1}H\}$ NMR spectrum of compound **3** in THF-D8 at 298 K.

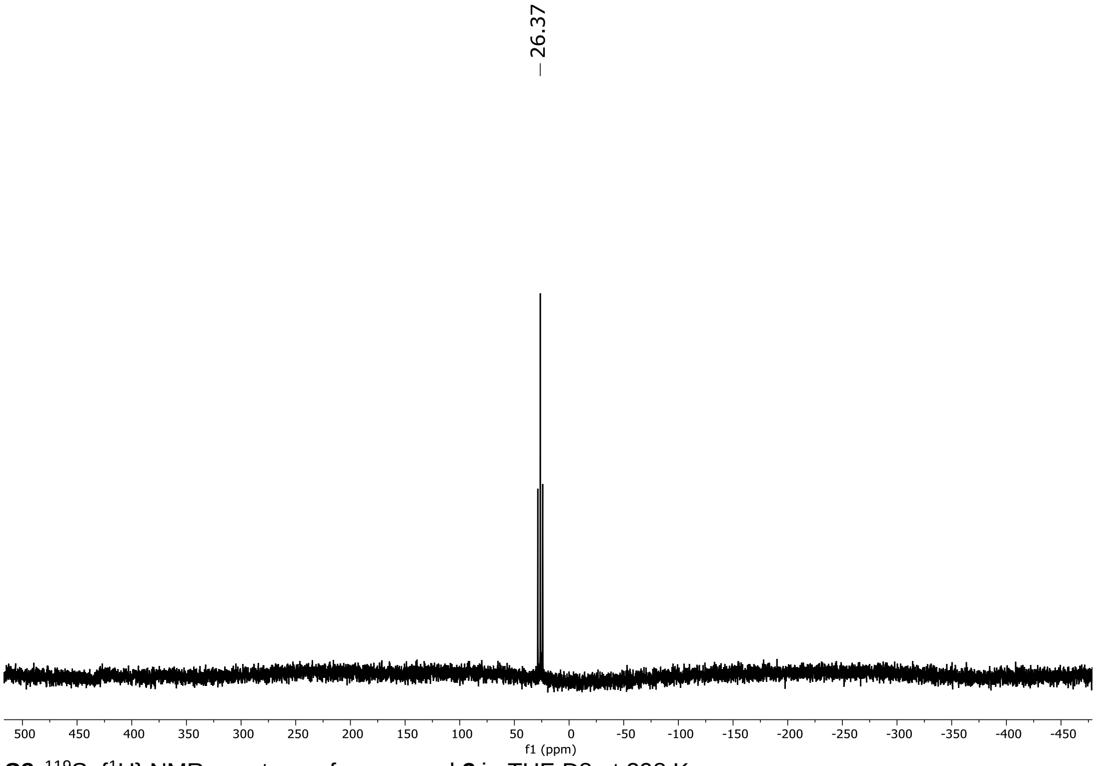

Figure S8. $^{119}Sn\{^{1}H\}$ NMR spectrum of compound **3** in THF-D8 at 298 K.

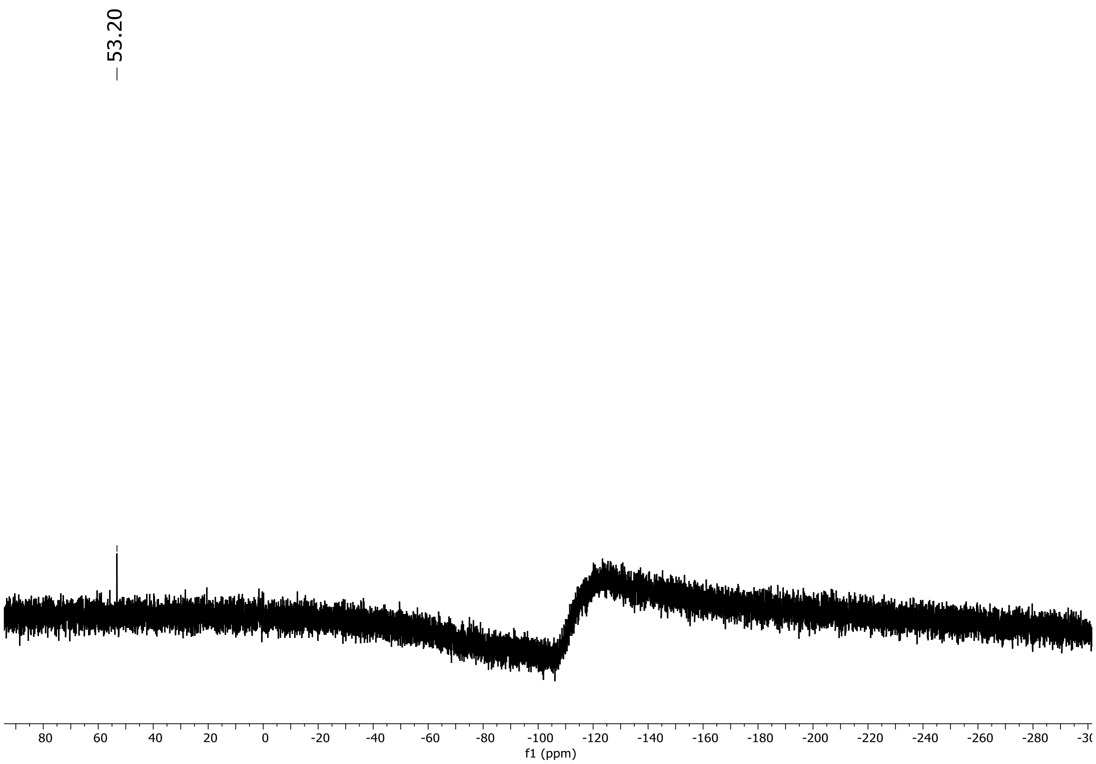

Figure S9. $^{29}Si\{^1H\}$ NMR spectrum of compound **3** in THF-D8 at 298 K.

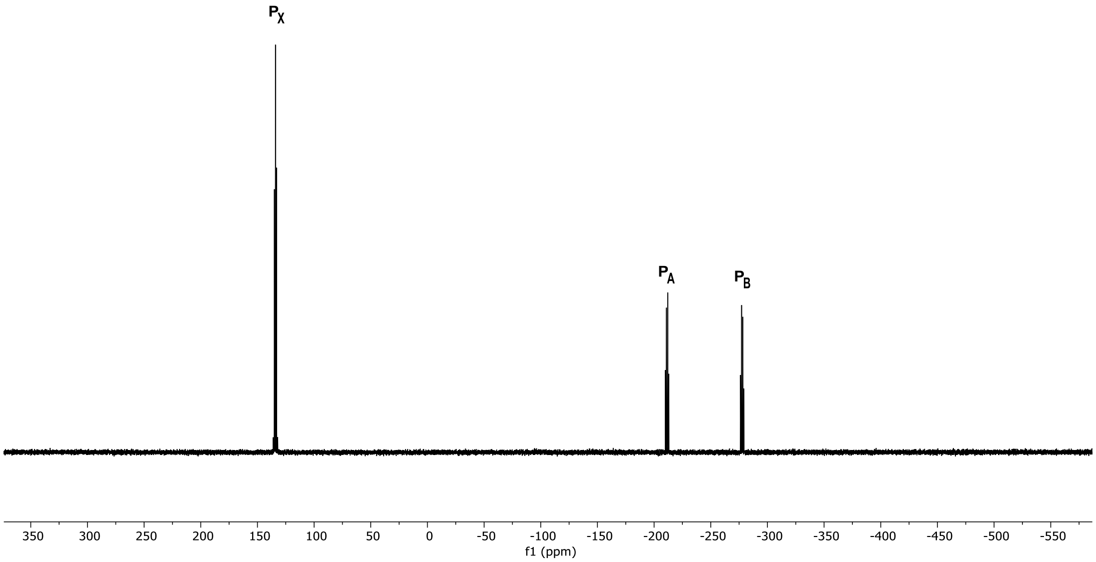

Figure S10. $^{31}P\{^1H\}$ NMR spectrum of compound **3** in THF-D8 at 298 K.

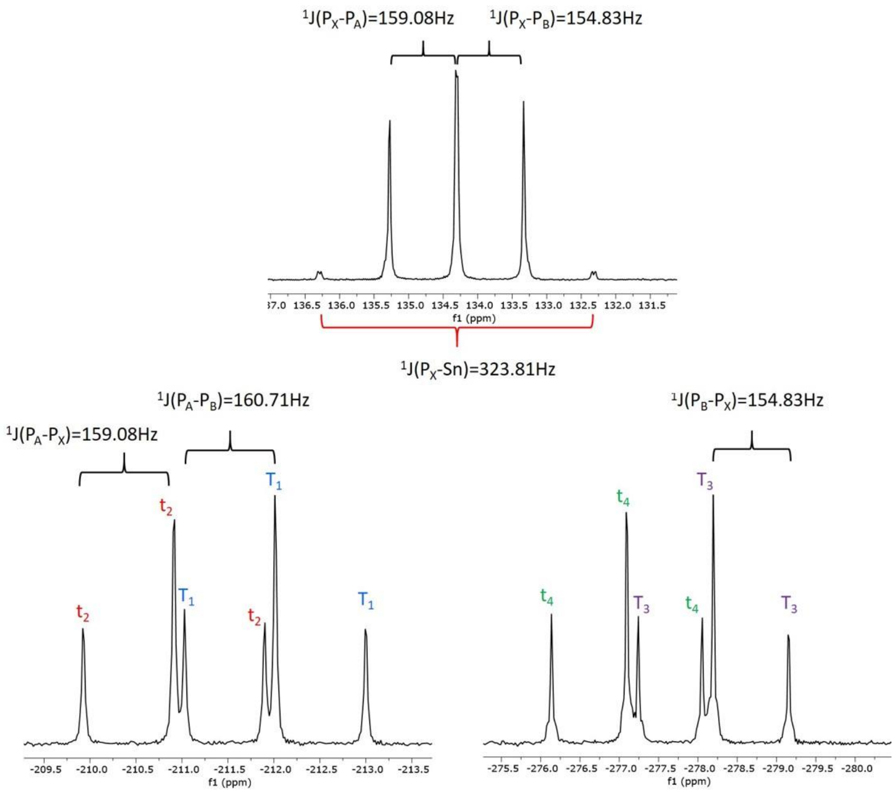

Figure S11. $^{31}P\{^1H\}$ NMR spectrum of compound **3** in THF-D8 at 298 K.

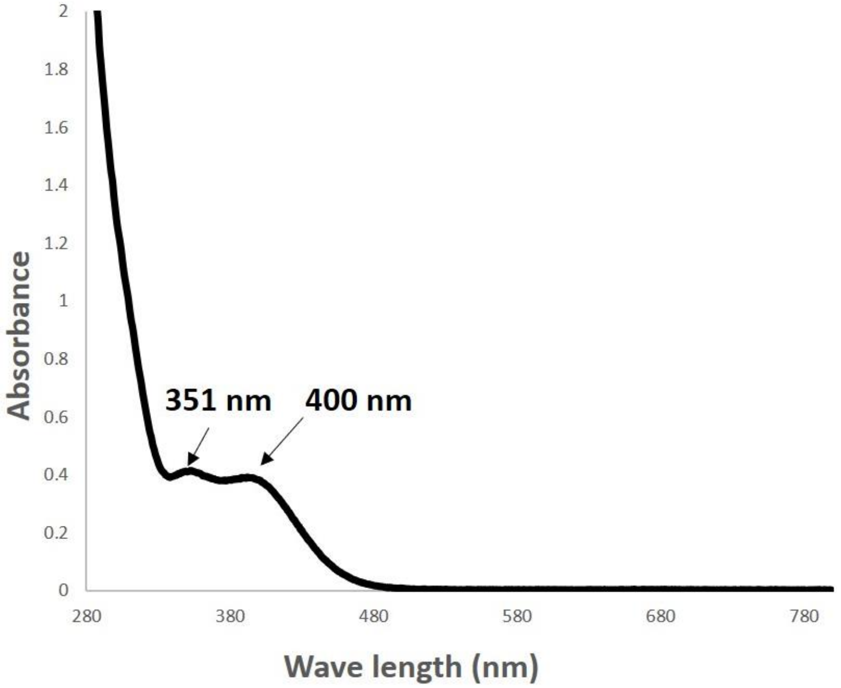

Figure S12. UV-VIS spectrum of compound **3** (THF, 2.5x10^{-3} M) at 298 K.

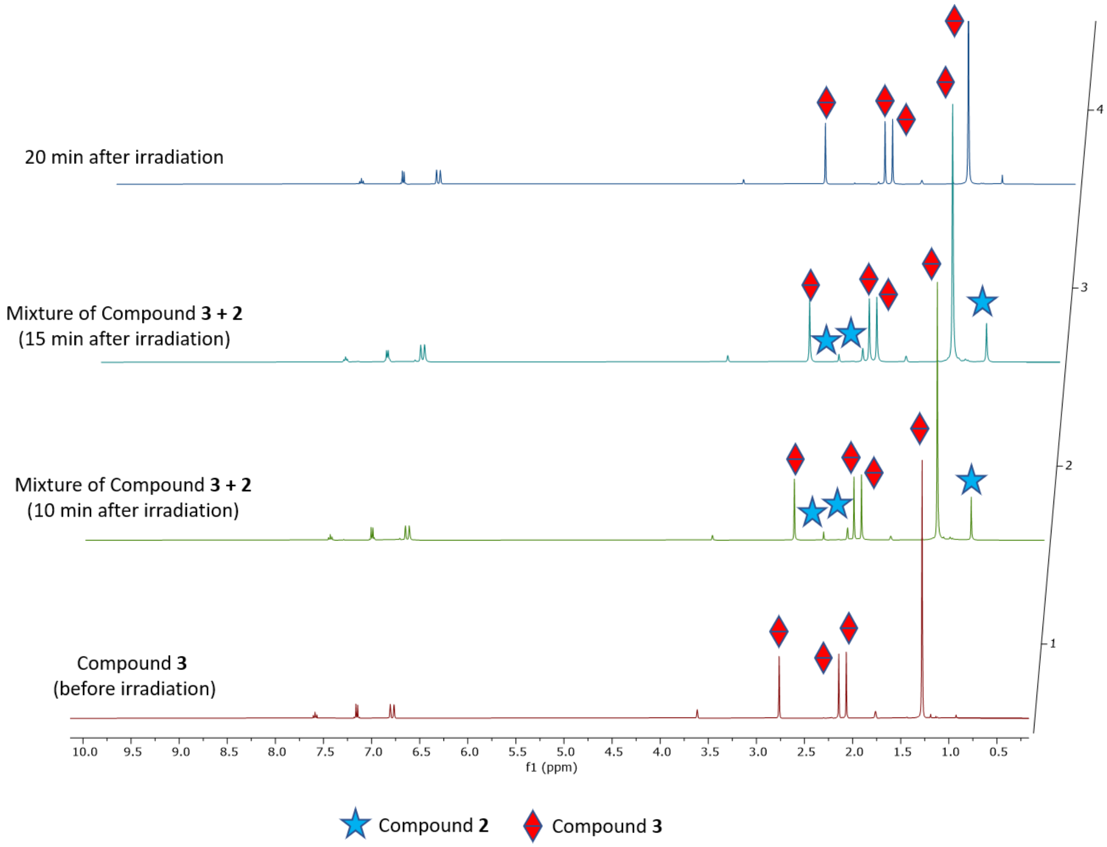

Figure S13. ^{1}H NMR spectrum of compound **3** before and after irradiation (UV 300-400 nm) (in THF-D8 at 298 K).

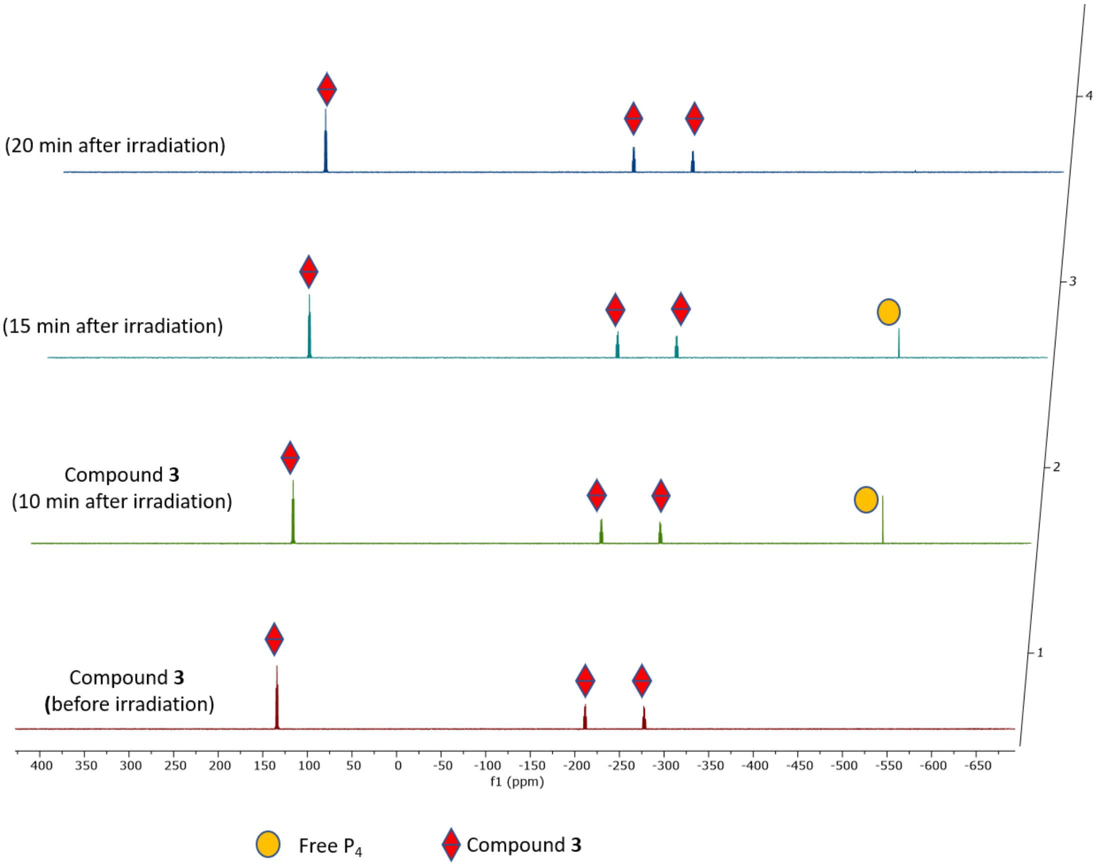

Figure S14. ^{31}P NMR spectrum of compound **3** before and after irradiation (UV 300-400 nm) (in THF-D8 at 298 K).

1.4 Preparation of compounds 4 and 5:

A THF solution (2 mL) of **3** (25 mg, 0.33 mmol, 1.00 eq) was directly added to a THF solution (3 mL) of ([PhN(C^{t}Bu)$_2$SiCl]) (30 mg, 0.99 mmol, 3.00 eq). The solution immediately turned from yellow to dark orange with the concomitant formation of a yellow precipitate. An NMR of the filtrate indicated the formation of compounds **4** (crude yield = 10%) and **5** (crude yield = 45%), on comparison to literature ^{31}P NMR values.[2] The crude yields were calculated based on integration in ^{31}P spectrum.
LIFDI-MS: calculated for **4 [$C_{45}H_{69}N_6P_3Si_3$] :** 870.41044, Found 870.41159. LIFDI-MS spectrum of compound **5** was not observed, but a known decomposition product[2b] of **5** was identified. [{PhC(NtBu)$_2$}SiP]$_2$ calculated m/z value 580.27362, Found 580.26964.

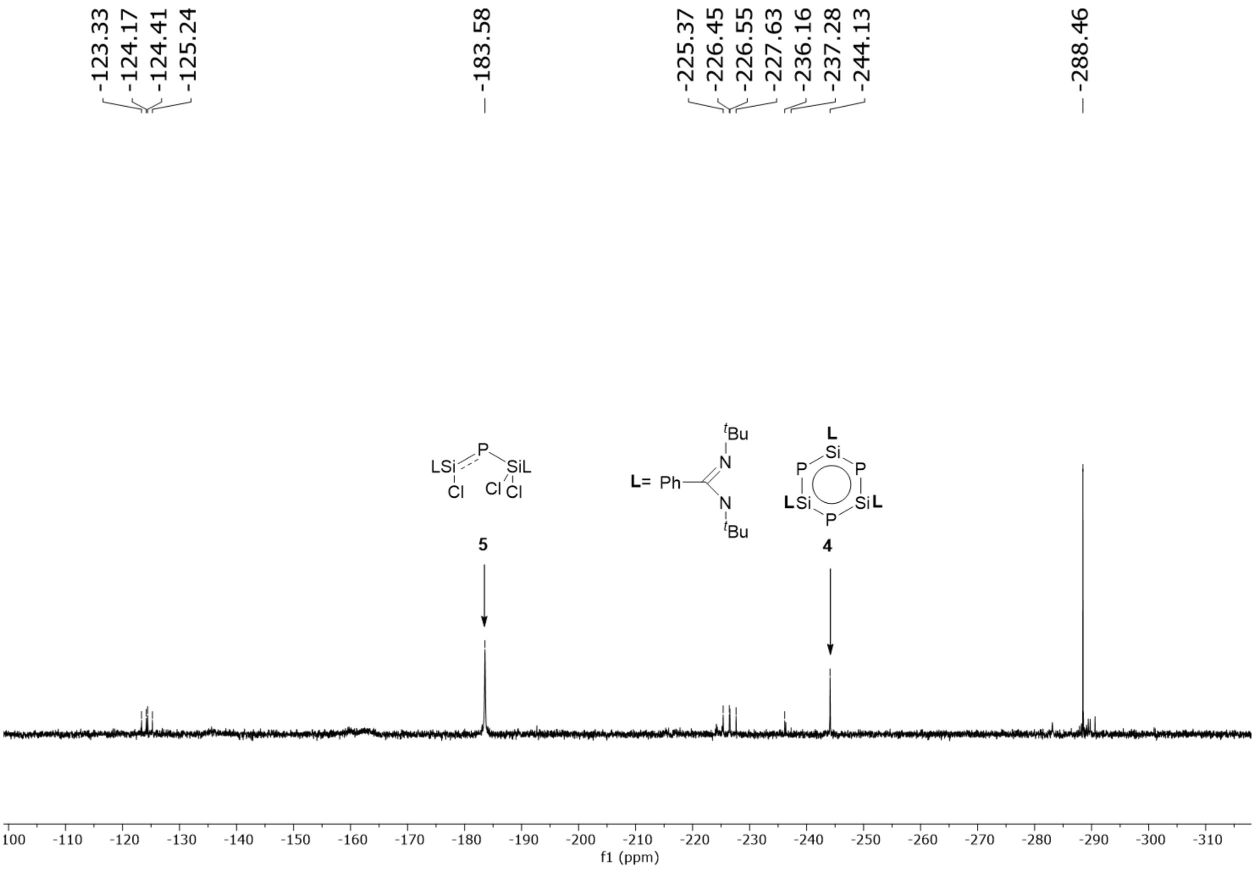

Figure S15. ^{31}P NMR spectrum of compound **4** and **5** (in THF-d_8 at 298 K).

2. X-Ray Crystallography

Data was collected using a single crystal X-ray diffractometer equipped with a CCD detector (Rigaku Oxford Diffraction, SuperNova, Atlas), a micro-focus sealed tube with CuKα radiation (λ = 1.54184Å) and a mirror monochromator using the CrysAlisPro software package (Compound 2) or equipped with a CMOS detector (Bruker APEX III, κ-CMOS), an IMS microsource with MoKα radiation (λ = 0.71073 Å) and a Helios optic using the APEX3 software package (Compound 3) or on a single crystal X-ray diffractometer.[3,4] The measurements were performed on single crystals coated with perfluorinated ether. The crystals were fixed on top of a micro sampler and frozen under a stream of cold nitrogen. A matrix scan was used to determine the initial lattice parameters. Reflections were corrected for Lorentz and polarisation effects, scan speed, and background using the CrysAlisPro software package or SAINT.[4,5] Absorption correction, including odd and even ordered spherical harmonics was performed using the CrysAlisPro software package or SADABS.[4,5] Space group assignment was based upon systematic absences, E statistics, and successful refinement of the structure. The structures were solved using SHELXT with the aid of successive difference Fourier maps, and were refined against all data using SHELXL-2014 in conjunction with SHELXLE.[6,7,8] Hydrogen atoms were calculated in ideal positions as follows: Methyl hydrogen atoms were refined as part of rigid rotating groups, with a C–H distance of 0.98 Å and Uiso(H) = 1.5·Ueq(C). Other H atoms were placed in calculated positions and refined using a riding model, with methylene and aromatic C–H distances of 0.99 Å and 0.95 Å, respectively, and other C–H distances of 1.00 Å, all with Uiso(H) = 1.2·Ueq(C). Non-hydrogen atoms were refined with anisotropic displacement parameters. Full-matrix least-squares refinements were carried out by minimizing Σw(Fo 2 - Fc 2)2 with the SHELXL weighting scheme.[6] Neutral atom scattering factors for all atoms and anomalous dispersion corrections for the non-hydrogen atoms were taken from International Tables for Crystallography.[9]

2.1 Refinement details for compound 2

CCDC number	2035171
Empirical formula	$C_{36}H_{526}Si_1Sn_1$
Formula weight	631.55
Temperature/K	150(10)
Crystal system	monoclinic
Space group	$P2_1/n$
a/Å	11.1308(3)
b/Å	18.2702(4)
c/Å	16.1897(4)
α/°	90
β/°	91.298(2)
γ/°	90
Volume/Å^3	3291.52(14)
Z	5
ρ_{calc}g/cm^3	1.593
μ/mm^{-1}	8.331
F(000)	1660.0
Crystal size/mm^3	0.18 × 0.15 × 0.08
Radiation	CuKα (λ = 1.54184)
2Θ range for data collection/°	7.296 to 147.674
Index ranges	$-13 \leq h \leq 13$, $-22 \leq k \leq 22$, $-3 \leq l \leq 19$
Reflections collected	6536
Independent reflections	6536 [R_{int} = 2.55, R_{sigma} = 0.0499]
Data/restraints/parameters	6536/0/359
Goodness-of-fit on F^2	1.051
Final R indexes [I>=2σ (I)]	R_1 = 0.0728, wR_2 = 0.2156
Final R indexes [all data]	R_1 = 0.0830, wR_2 = 0.2326
Largest diff. peak/hole / e Å-3	3.50/-2.65
Comments:	Compound 2 was refined according to the twin law found with platon, remaining electron density due to lone pair of the tin centre

2.2 Refinement details for compound 3

CCDC Number	2035172
Empirical formula	$C_{36}H_{51}P_4SiSn$
Formula weight	754.42
Temperature/K	100.02
Crystal system	orthorhombic
Space group	$Pna2_1$
a/Å	19.2663(11)
b/Å	11.4979(8)
c/Å	16.5652(11)
α/°	90
β/°	90
γ/°	90
Volume/Å^3	3669.6(4)
Z	4
ρ_{calc}g/cm^3	1.366
μ/mm^{-1}	0.927

F(000)	1564.0
Crystal size/mm^3	0.22 × 0.2 × 0.1
Radiation	MoKα (λ = 0.71073)
2Θ range for data collection/°	4.126 to 51.406
Index ranges	$-22 \leq h \leq 23$, $-14 \leq k \leq 14$, $-20 \leq l \leq 20$
Reflections collected	156239
Independent reflections	6967 [R_{int} = 0.0320, R_{sigma} = 0.0092]
Data/restraints/parameters	6967/1/395
Goodness-of-fit on F^2	1.037
Final R indexes [I>=2σ (I)]	R_1 = 0.0133, wR_2 = 0.0340
Final R indexes [all data]	R_1 = 0.0138, wR_2 = 0.0348
Largest diff. peak/hole / e Å^{-3}	0.27/-0.34
Comments	Compound 3 was refined as an inverted twin

3. Computations

3.1 General

All calculations were performed with ORCA 4.0.1.[10] The geometric parameters were optimized at the PBE0/def2-SVP level of theory[11] with tighter than default convergence- (tightscf, tightopt) and finer than default grid values (grid5, finalgrid6, gridx4) without symmetry- or internal coordinate constraints. They were verified as true minima by the absence of negative eigenvalues in the harmonic vibrational frequency analysis.[12] For tin, the small-core (28 electron) def2-ECP relativistic pseudopotential was used.[13] The D3 dispersion correction with Becke–Johnson (BJ) damping was applied.[14] In all calculation, the resolution of identity and chain of spheres approximation (RIJCOSX) and the related auxiliary basis sets (def2/J) were used.[11] Single-point calculations were performed at the PBE0/def2-TZVPP//PBE0/def2-SVP level of theory[15] for the calculation of the energies of the HOMO and LUMO given in Fig. 2. The energies given in Fig. 2 ($\Delta E^{s/t}$) and Fig. 4 were refined with single-point calculations at the DLPNO-CCSD(T)/def2-TZVPP//PBE0/def2-SVP level of theory using the default “normalPNO” settings,[16] the RIJCOSX approximation, as well as the auxiliary basis sets def2/J and def2-TZVPP/C.[17] Note that the SMD module as currently implemented in ORCA is not applicable with the DLPNO-CCSD(T) calculations, because it currently relates only to the HF orbitals. Solvation effects were hence included by single-point calculations with the PBE0 functional at the PBE0(SMD=THF)/def2-TZVPP//PBE0/def2-SVP level of theory[18] as has been suggested by Neese et al..[19] Calculated structures and molecular orbitals were visualized with Chemcraft, Avogadro[20] and IBOView[21].
For the calculation of the NMR shifts of tin, various functionals (PBE0, TPSS, TPSSh, M06), quasi-relativic core potentials such as SDD, and scalar relativistic calculations using the Zeroth Order Regular Approximation (ZORA)[22] or Douglas-Kroll-Hess method (DKH2)[23] were evaluated. However, basis-set effects, i.e. (de-)contraction, choice of basis set, etc., evolved to be more important than the choice of the method, and the best qualitative agreement with the experiment was obtained with the def2-ECP. Further, note that the Ahlrichs all-electron def2-XVP basis set is only a valence basis set and comes with the def2-ECP for the core electrons in case of tin. We hence attribute the deviation of the calculated tin NMR shifts to inadequate/missing treatment of generally relativistic and in particular spin-orbit coupling effects as well as limitations of available basis sets / effective core potentials.[24]
The TD-DFT calculations, where 50 roots were calculated using the Tamm-Dancoff approximation and the def2/TZVPP basis set, were conducted with the functionals B3LYP, cam-B3LYP, PBE0, M06, M06-2X and ωB97-XD3. All functionals gave similar results and overestimate the experimentally observed transitions between approximately 6400 cm^{-1} (ωB97-XD3) and 7400 cm^{-1} (M06-2X) for the S_1 state (Table S1). Inclusion of implicit solvation did only have a marginal effect on the calculated transitions. Attempted explicit solvation through coordination of a THF molecule led to dissociation.

	S_1 in [nm]	S_2 in [nm]	S_3 in [nm]
exp	400	351	<351
camB3LYP	562	516	421
M062X	586	530	441
M06	614	565	507
PBE0	592	556	482

ωB97-XD3	551	507	408
ωB97-XD3 (SMD=THF)	572	509	423

Table S1. Calculated transitions to S_1, S_2 and S_3 states of **3** using various methods.

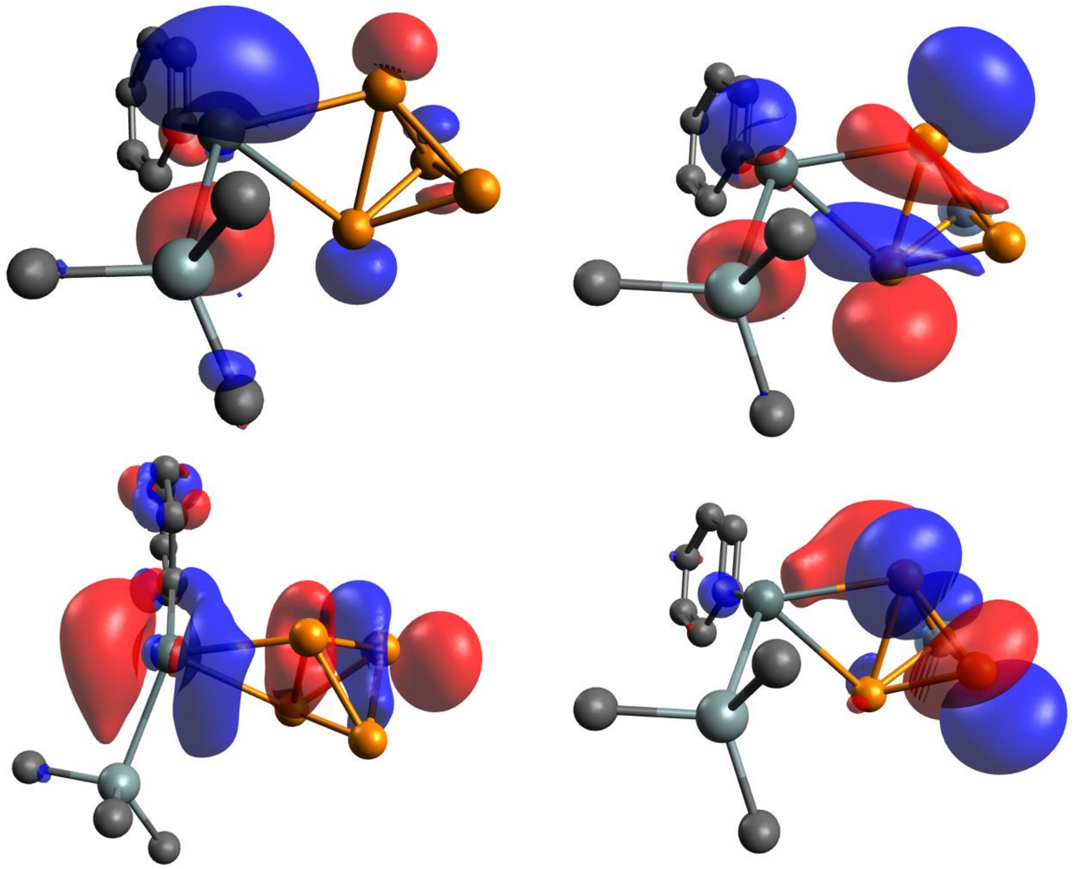

Figure S16. Frontier orbitals (up left: HOMO; up right: LUMO; down left: LUMO+1; down right: LUMO+2) of **3** as obtained with the ωB97-XD3 functional. Mesityl- and methyl substituents as well as hydrogen atoms are omitted for clarity.

Transition	f^{osc}	Character
$S_0 \rightarrow S_1$	0.18	84% HOMO → LUMO
		10% HOMO → LUMO+1
$S_0 \rightarrow S_2$	0.11	9% HOMO → LUMO
		79% HOMO → LUMO+1
$S_0 \rightarrow S_3$	0.03	2% HOMO → LUMO+1
		81% HOMO → LUMO+2

Figure S17. Character of transitions to S_1 and S_2 states of **3** as obtained with the ωB97-XD3 functional.

3.2 Energies

**Arylsilyl stannylene**	**Imag.**	***E*(SVP)**	***G*(SVP)**	***E*(TZVPP)**	***E*(TZVPP, SMD)**	***E*(DLPNO-CCSDT)**
P_4	n.a.	-1364.53452	-1364.55555	-1365.01674	-1365.01426	-1363.67316
**2**	n.a.	-1904.22800	-1903.51008	-1905.80001	-1905.82100	-1903.31888
σ-complex	n.a.	-3268.78107	-3268.06372	-3270.83206	-3270.85008	-3267.00692
TS	-77	-3268.75742	-3268.03765	-3270.80708	-3270.82704	-3266.98032
**3**	n.a.	-3268.81346	-3268.08937	-3270.85801	-3270.88062	-3267.03130

Table S2. Imaginary frequency of transition state (value given in cm^{-1}) as well as calculated energies of compounds for P_4 activation by arylsilyl stannylene **2** (values given in [E_h]).

**Diarylstannylene**	**Imag.**	***E*(SVP)**	***G*(SVP)**	***E*(TZVPP)**	***E*(TZVPP, SMD)**	***E*(DLPNO-CCSDT)**
P_4	n.a.	-2070.20211	-2069.44178	-2072.13668	-2072.16814	-2069.47346
Stannylene_diaryl	n.a.	-3434.76008	-3433.99683	-3437.17091	-3437.19970	-3433.16299
σ-complex_diaryl	n.a.	-3434.73254	-3433.96772	-3437.14063	-3437.17026	-3433.13120
TS_diaryl	-181	-3434.77820	-3434.01106	-3437.18431	-3437.21619	-3433.17651
Product_diaryl	n.a.	-2070.20211	-2069.44178	-2072.13668	-2072.16814	-2069.47346

Table S3. Imaginary frequency of transition state (value given in cm^{-1}) as well as calculated energies of compounds for P_4 activation by the diaryl stannylene **(^{Mes}Ter)$_2$Sn** (values given in [E_h]).

3.3 XYZ Coordinates

P_4

P	9.25127	9.54722	9.16762
P	11.08586	9.33294	7.97169
P	9.16929	9.76769	6.97973
P	10.14907	11.31099	8.20642

Stannylene 2

Sn	6.34457	10.98655	4.07856
Si	9.05225	11.02506	4.03607
C	10.88273	9.70604	5.89689
H	10.81096	8.74005	5.37762
H	11.15699	9.48799	6.94570
H	11.71857	10.26555	5.45103
C	8.49720	9.81159	1.49608
H	7.41753	9.91267	1.70888
H	8.59587	8.95812	0.79984
H	8.80527	10.71461	0.95569
C	6.86903	12.94137	-0.28330
H	7.66024	13.37462	-0.90272
C	5.09093	13.59184	5.40692
C	6.30470	11.71709	-0.65644
C	4.37259	14.79237	5.44804
H	4.07013	15.20654	6.41415
C	7.21041	14.53445	7.20548
H	7.45531	14.62931	6.13907
H	8.13987	14.59398	7.78889
H	6.59664	15.41225	7.46523
C	4.65330	11.70054	6.99824
C	6.79466	12.49075	8.60914
H	7.62419	12.81165	9.24594
C	5.49116	13.04966	4.17267
C	9.32354	9.54814	2.76263
C	5.41479	12.84079	6.65390
C	10.78990	9.36176	2.35806
H	11.19349	10.24117	1.83570
H	10.88214	8.50499	1.66498
H	11.44163	9.15688	3.21958
C	5.46773	13.05710	1.68684
C	6.81813	10.97085	-1.85167
H	7.73300	10.41152	-1.59302
H	7.07671	11.65436	-2.67389
H	6.08268	10.24390	-2.22404
C	5.10558	13.69793	2.98398
C	7.09727	14.94204	1.21240
H	6.38957	15.76928	1.04448
H	7.98582	15.13024	0.59453
H	7.39090	14.99030	2.27007
C	4.82270	11.86615	1.28191
C	11.49432	12.60732	3.77847
H	11.70432	12.55900	4.85619
H	11.97500	13.52721	3.39783
H	12.00021	11.75522	3.30160
C	4.38217	14.89369	3.03786
H	4.08240	15.38623	2.10865
C	8.45513	9.64261	6.48181
H	7.52253	10.21987	6.61407
H	8.75754	9.31357	7.49354
H	8.21658	8.74122	5.90124
C	4.02543	15.44499	4.26702
H	3.45736	16.37746	4.30385

C	9.56927	10.49448	5.85845
C	3.42114	11.32429	6.22188
H	2.81845	12.21218	5.98191
H	2.80028	10.62188	6.79466
H	3.67164	10.83927	5.26391
C	9.36141	13.85474	4.16081
H	8.27429	13.89540	3.98888
H	9.80444	14.78734	3.76535
H	9.52302	13.85083	5.24584
C	6.46752	13.26107	7.49122
C	9.99356	12.64247	3.46771
C	6.47224	13.62035	0.86832
C	9.82634	12.84364	1.95980
H	10.38378	12.10058	1.37238
H	10.21446	13.83815	1.67426
H	8.77352	12.79989	1.64912
C	3.58170	11.35610	1.96487
H	3.56102	10.25762	1.99439
H	2.69377	11.69036	1.40162
H	3.47758	11.73513	2.98871
C	8.80976	8.22755	3.35076
H	9.38672	7.89467	4.22456
H	8.88647	7.43082	2.58840
H	7.75021	8.28949	3.64688
C	5.02671	10.95266	8.11962
H	4.44304	10.06414	8.37789
C	6.53635	10.45880	10.07718
H	7.00147	11.05492	10.87556
H	7.28102	9.71512	9.74607
H	5.69007	9.90465	10.50833
C	5.27180	11.20969	0.12727
H	4.78106	10.27847	-0.17124
C	6.10712	11.31690	8.92465
C	9.72657	11.72734	6.75292
H	9.89716	11.40672	7.79691
H	8.82137	12.34908	6.75272
H	10.58024	12.35600	6.46523

σ-complex

Sn	7.26065	8.83697	8.05282
P	10.63786	9.63993	9.01114
P	12.42967	10.69036	8.22097
P	10.86445	9.93188	6.85801
P	10.44136	11.64569	8.15844
Si	6.97371	6.16376	7.80305
C	8.24293	4.04897	9.35738
H	7.54855	3.32526	8.90548
H	8.52743	3.65404	10.35016
H	9.15536	4.06431	8.74469
C	3.16256	11.64835	8.70114
H	2.31480	12.32190	8.84834
C	7.37526	8.75832	12.83332
C	8.26719	8.15463	13.87713
H	8.90995	7.37557	13.43323
H	7.68486	7.67934	14.67955
H	8.93004	8.90599	14.32982
C	3.93561	8.12225	11.31762
H	3.15079	8.78723	11.71349
H	3.83046	7.14901	11.81700
H	3.72708	7.99453	10.24722
C	7.61404	5.43836	9.51223
C	3.04054	8.71816	5.47452
H	3.05087	8.37926	6.51992
H	2.70216	7.88584	4.84229
H	2.28495	9.51745	5.41046
C	4.38909	9.21421	5.03619
C	9.71262	6.20080	6.88183
H	10.05609	5.48566	7.64188
H	9.79529	7.21275	7.30694
H	10.42565	6.14891	6.03897
C	3.56656	11.30775	7.41153
H	3.04446	11.72496	6.54601
C	8.64702	6.39086	10.13002
H	9.51233	6.57920	9.48094
H	9.03051	5.96280	11.07496
H	8.20360	7.36773	10.39217
C	5.13817	10.09440	5.84917
C	8.01634	6.89682	5.24244
H	7.02863	6.77282	4.78198
H	8.77409	6.78793	4.44465
H	8.08705	7.94602	5.58421
C	4.65350	10.44903	7.21421
C	5.23193	5.42458	7.31357
C	4.94603	10.29343	9.61876
C	7.41035	11.66956	10.37644
H	6.65851	12.45945	10.52892
H	7.51202	11.53439	9.28911
H	8.37562	12.02679	10.75955
C	6.35944	10.62005	5.37008
C	6.84965	10.17859	4.13313
H	7.80404	10.57701	3.77551
C	7.78746	9.86107	12.08220
H	8.75153	10.32926	12.30070
C	4.90856	8.82002	3.80369
H	4.32979	8.12555	3.18675
C	6.15142	9.26858	3.34361
C	4.91836	5.74598	5.85032
H	5.01668	6.81791	5.63141
H	3.87576	5.45717	5.62434
H	5.56349	5.19787	5.14973
C	7.08665	11.73332	6.07519
H	6.88558	12.68716	5.55906
H	8.17486	11.57697	6.06422
H	6.76416	11.85308	7.11673
C	8.29307	4.47846	5.79564
H	8.50093	3.71543	6.56010
H	9.07614	4.38739	5.02041
H	7.33742	4.22095	5.31714
C	3.85519	11.14907	9.80302
H	3.55832	11.43546	10.81607
C	6.46098	5.33568	10.51432
H	5.94163	6.29565	10.63738
H	6.85804	5.05420	11.50665
H	5.71938	4.57476	10.23505
C	5.33310	9.91203	8.32224
C	4.14579	6.09025	8.16529
H	4.23009	5.84057	9.23035
H	3.14692	5.75735	7.82819
H	4.18358	7.18746	8.07752
C	5.29720	8.71028	11.54936
C	6.71859	8.76040	2.05129
H	7.46451	9.45236	1.63527
H	5.93229	8.60332	1.29822
H	7.22103	7.79048	2.20471

C	5.75395	9.78914	10.76695
C	6.11518	8.21768	12.56890
H	5.76091	7.37210	13.16572
C	8.29851	5.89805	6.37325
C	6.99228	10.40279	11.06740
C	5.15736	3.90446	7.49556
H	5.91685	3.37471	6.90238
H	4.16860	3.53808	7.16343
H	5.27681	3.59691	8.54387

TS

Sn	7.03640	8.29095	8.99921
P	9.28297	9.58002	10.00352
P	11.08277	9.05942	8.83103
P	9.30283	9.44633	7.59460
P	10.25637	11.10201	8.71360
Si	7.50756	6.01546	7.56189
C	8.86798	3.64009	8.55082
H	8.07207	3.02045	8.11478
H	9.27828	3.08393	9.41344
H	9.67578	3.72170	7.80959
C	3.77051	12.12639	8.48150
H	3.01532	12.91629	8.46624
C	6.96298	9.79351	13.58230
C	7.59867	9.45613	14.89958
H	8.40835	8.71868	14.76744
H	6.87038	9.01929	15.59850
H	8.04007	10.34425	15.37515
C	4.15670	8.48821	11.36308
H	3.36303	9.10819	10.92121
H	3.72384	7.88357	12.17177
H	4.49381	7.79448	10.57231
C	8.36594	5.01169	9.02181
C	3.48819	8.75708	5.75622
H	3.60442	8.35136	6.77142
H	3.04091	7.98018	5.12131
H	2.76823	9.58702	5.83820
C	4.79562	9.23774	5.19172
C	10.08735	6.16604	6.25265
H	10.51255	5.32658	6.82038
H	10.28584	7.09076	6.81179
H	10.65132	6.23531	5.30543
C	4.23323	11.57278	7.29485
H	3.85515	11.93794	6.33563
C	9.55727	5.79291	9.58824
H	10.32992	6.01278	8.84035
H	10.03619	5.20811	10.39412
H	9.23802	6.74576	10.03974
C	5.62641	10.09046	5.94172
C	8.17074	7.12853	5.02300
H	7.14444	7.02255	4.65383
H	8.83685	7.17584	4.14208
H	8.22626	8.10431	5.52740
C	5.19286	10.54908	7.29193
C	5.69984	5.32509	7.22847
C	5.26868	10.66756	9.70954
C	7.39353	12.32528	10.76869
H	6.67760	13.16052	10.84388
H	7.49914	12.10833	9.69573
H	8.36325	12.67484	11.14975
C	6.76512	10.66458	5.34469
C	7.11523	10.28453	4.04674
H	8.01155	10.71911	3.59368
C	7.44821	10.83747	12.79474
H	8.27738	11.44837	13.16468
C	5.17349	8.89353	3.89117
H	4.53199	8.21841	3.31614
C	6.34208	9.38749	3.30733
C	5.19389	5.80749	5.86785
H	5.29836	6.89292	5.75094
H	4.12237	5.55927	5.76280
H	5.71825	5.33112	5.02875
C	7.52158	11.76383	6.03364
H	7.00558	12.72684	5.87922
H	8.54270	11.86282	5.63996
H	7.57751	11.61475	7.11972
C	8.46431	4.65387	5.17044
H	8.77539	3.78527	5.76836
H	9.11452	4.68387	4.27703
H	7.44198	4.46868	4.81417
C	4.29929	11.67866	9.68516
H	3.97428	12.12032	10.63130
C	7.39292	4.79536	10.18786
H	7.00336	5.74872	10.58066
H	7.92315	4.29424	11.01765
H	6.53906	4.15835	9.92088
C	5.71602	10.05906	8.50873
C	4.72868	5.87140	8.28267
H	5.00789	5.61050	9.31254
H	3.71525	5.46859	8.10348
H	4.64162	6.96988	8.22186
C	5.30286	9.31797	11.85528
C	6.76550	8.95309	1.93484
H	7.25435	9.77067	1.38446
H	5.91011	8.60194	1.33981
H	7.48882	8.12215	1.99473
C	5.84886	10.33622	11.04823
C	5.87343	9.06096	13.10126
H	5.45681	8.25769	13.71644
C	8.60041	5.97687	5.93680
C	6.90089	11.13464	11.54244
C	5.63312	3.79299	7.24233
H	6.31830	3.33780	6.51254
H	4.61024	3.46899	6.97631
H	5.85844	3.36365	8.22817

3

Sn	7.54627	8.83287	8.05144
P	9.44296	9.58291	9.63627
P	10.90752	9.26039	7.97117
P	9.32556	9.89688	6.51405
P	9.97946	11.21866	8.20397
Si	7.49379	6.17225	7.72094
C	8.55323	3.91877	9.18750
H	7.71972	3.31640	8.80126
H	8.84524	3.48653	10.16132
H	9.40933	3.78493	8.51045
C	3.55411	11.85939	8.62743
H	2.70202	12.53209	8.75165
C	7.29048	9.05094	13.14408
C	8.05581	8.49411	14.30776
H	8.86047	7.82658	13.95564
H	7.40864	7.90626	14.97503
H	8.52945	9.29058	14.90005

C	4.17017	8.22151	11.13692
H	3.34287	8.68160	11.70346
H	4.16497	7.14736	11.37131
H	3.93886	8.35437	10.07387
C	8.19402	5.39944	9.37956
C	3.54037	8.79470	5.54086
H	3.59382	8.48195	6.59245
H	3.22881	7.93279	4.93596
H	2.74109	9.55139	5.48095
C	4.83772	9.36901	5.04557
C	10.11640	5.91397	6.53922
H	10.44049	5.16951	7.27950
H	10.36599	6.91271	6.92593
H	10.72753	5.76052	5.63250
C	4.00352	11.52165	7.35610
H	3.51146	11.93113	6.46984
C	9.44759	6.12819	9.87679
H	10.24493	6.20497	9.12709
H	9.86048	5.58481	10.74555
H	9.21959	7.14624	10.22440
C	5.53317	10.33368	5.79997
C	8.35908	6.75132	5.01968
H	7.31497	6.75609	4.68477
H	8.98502	6.46870	4.15430
H	8.62573	7.78917	5.27187
C	5.09344	10.65988	7.18551
C	5.65095	5.61466	7.39579
C	5.30088	10.47900	9.59832
C	7.45366	12.03541	10.78234
H	6.72515	12.81428	11.06322
H	7.47627	12.01046	9.68406
H	8.44371	12.34847	11.14067
C	6.60725	11.03925	5.22199
C	7.07296	10.64823	3.96400
H	7.93077	11.17297	3.53430
C	7.70083	10.22486	12.50826
H	8.56182	10.77408	12.89902
C	5.33311	9.01145	3.78849
H	4.80786	8.24280	3.21323
C	6.47379	9.60961	3.24778
C	5.24729	5.92326	5.95297
H	5.43706	6.97055	5.68240
H	4.16515	5.74132	5.83024
H	5.76733	5.28898	5.22299
C	7.17262	12.25527	5.89841
H	6.47198	13.10029	5.78857
H	8.13533	12.54913	5.45920
H	7.31614	12.10758	6.97740
C	8.40473	4.34678	5.68020
H	8.58016	3.59300	6.46076
H	9.11059	4.13463	4.85755
H	7.39337	4.18987	5.28142
C	4.20391	11.33843	9.74000
H	3.86823	11.59925	10.74737
C	7.15150	5.51848	10.49689
H	6.83199	6.56039	10.65628
H	7.59513	5.17353	11.44788
H	6.25778	4.90510	10.31785
C	5.75922	10.12435	8.31044
C	4.71377	6.40104	8.31304
H	4.90831	6.21388	9.37442
H	3.66431	6.11698	8.11494
H	4.79572	7.48625	8.14920
C	5.47003	8.86464	11.52565
C	7.04414	9.14857	1.93910
H	7.48898	9.98194	1.37563
H	6.27880	8.67462	1.30755
H	7.84244	8.40560	2.10641
C	5.96550	9.98922	10.84039
C	6.15571	8.40390	12.65405
H	5.78200	7.51433	13.17060
C	8.63414	5.78231	6.17803
C	7.05065	10.71520	11.37397
C	5.43248	4.11630	7.64164
H	6.08110	3.48532	7.01737
H	4.38765	3.85699	7.39281
H	5.59112	3.83067	8.69090

Stannylene_diaryl

Sn	7.98726	7.77233	8.57795
C	5.50682	12.01789	7.63601
H	5.09041	13.02208	7.52760
C	6.20452	8.45256	12.98469
C	6.21095	7.82298	14.34669
H	6.48214	6.75551	14.28292
H	5.21885	7.87642	14.81929
H	6.93681	8.30733	15.01517
C	3.91158	8.62187	9.94112
H	3.58586	9.58382	9.51859
H	3.07374	8.17556	10.49077
H	4.14192	7.96245	9.08977
C	4.50840	8.28248	5.39398
H	4.65816	7.82570	6.38339
H	3.87415	7.61693	4.79333
H	3.95724	9.22022	5.56779
C	5.81518	8.55689	4.70832
C	5.90487	11.29800	6.51603
H	5.79457	11.73185	5.51793
C	6.79170	9.34730	5.34179
C	6.44985	10.00875	6.63242
C	6.16969	10.15385	9.02623
C	8.44241	10.66628	10.83650
H	8.09058	11.66139	10.52519
H	8.96701	10.23061	9.96890
H	9.17334	10.79112	11.64704
C	7.98966	9.66517	4.67551
C	8.17929	9.20058	3.37224
H	9.10364	9.46253	2.84785
C	7.28132	9.22123	12.53803
H	8.12854	9.39710	13.20753
C	6.05559	8.09183	3.41205
H	5.29287	7.48049	2.91992
C	7.22557	8.41384	2.72150
C	9.03370	10.51248	5.34562
H	9.89484	10.67924	4.68380
H	9.40693	10.03315	6.26497
H	8.62882	11.49197	5.64246
C	5.63209	11.43767	8.89401
H	5.30251	11.97354	9.78884
C	6.59714	9.41230	7.89926
C	5.10441	8.80204	10.83322
C	7.47807	7.88981	1.33799
H	8.00459	8.62881	0.71564
H	6.54129	7.61914	0.83028

H	8.10749	6.98349	1.36881
C	6.21720	9.54980	10.39137
C	5.11750	8.27387	12.12489
H	4.24771	7.70911	12.47129
C	7.29952	9.79460	11.26359
C	7.69742	5.70222	7.73506
C	8.92024	5.22498	7.20892
C	6.68828	4.76093	8.01064
C	9.12555	3.86486	6.96205
C	6.90535	3.39887	7.74758
C	8.11538	2.94698	7.23346
H	10.08312	3.53300	6.55017
H	6.10587	2.68653	7.97013
H	8.27127	1.88134	7.04789
C	9.99718	6.20012	6.86344
C	11.00000	6.54502	7.79479
C	10.02425	6.73974	5.55966
C	11.95325	7.50221	7.43357
C	11.00462	7.67891	5.23769
C	11.96203	8.09319	6.16870
H	12.71893	7.78474	8.16198
H	11.02103	8.09684	4.22721
C	5.36302	5.10734	8.59859
C	4.22867	5.14411	7.76237
C	5.21654	5.23706	9.99193
C	2.96860	5.32657	8.33516
C	3.93156	5.37543	10.52596
C	2.79647	5.43029	9.71776
H	2.09146	5.36550	7.68158
H	3.81811	5.44275	11.61186
C	9.03858	6.26579	4.53488
H	9.28591	6.65535	3.54043
H	9.01703	5.16720	4.48973
H	8.01617	6.59247	4.77881
C	11.05502	5.88250	9.13909
H	11.07529	4.78679	9.04026
H	11.94176	6.20245	9.70320
H	10.16442	6.12975	9.74140
C	12.96438	9.15292	5.81579
H	13.82395	9.14238	6.50079
H	13.34069	9.02744	4.78948
H	12.50843	10.15587	5.87304
C	1.43145	5.63565	10.30627
H	0.68259	4.98644	9.82811
H	1.42155	5.43250	11.38674
H	1.09409	6.67638	10.16195
C	4.36092	4.92696	6.28179
H	5.23026	5.45579	5.86616
H	4.50621	3.85808	6.05511
H	3.45831	5.26055	5.75137
C	6.40944	5.21580	10.90492
H	6.11516	4.96739	11.93448
H	7.16953	4.49584	10.57000
H	6.89632	6.20681	10.95094

σ-complex_diaryl

Sn	8.07453	7.95337	8.47734
P	8.21744	6.60873	11.37807
P	9.41774	4.77486	11.29745
P	10.16096	6.55292	12.37696
P	8.47195	5.39078	13.19422
C	4.77005	11.68889	7.80382
H	4.14908	12.58391	7.71879
C	6.77842	8.80134	13.19557
C	7.05572	8.40745	14.61630
H	8.13034	8.24024	14.78394
H	6.51943	7.48875	14.89282
H	6.73623	9.20058	15.31306
C	4.08542	8.29525	10.54388
H	3.45991	9.16383	10.28561
H	3.50288	7.62436	11.18714
H	4.29260	7.76906	9.60095
C	4.17997	8.62160	4.92984
H	4.06012	8.35016	5.98770
H	3.70108	7.85033	4.31025
H	3.62038	9.55910	4.77921
C	5.62436	8.79225	4.55101
C	5.25191	11.05861	6.66377
H	5.01912	11.46694	5.67650
C	6.54896	9.37896	5.44328
C	6.05943	9.91321	6.74761
C	5.91505	10.04168	9.15785
C	8.33504	11.01756	10.51381
H	7.80055	11.92439	10.19209
H	8.77725	10.57249	9.60704
H	9.15702	11.31271	11.17967
C	7.87402	9.60871	5.02337
C	8.24524	9.24979	3.72347
H	9.26363	9.46979	3.39258
C	7.65494	9.64368	12.50550
H	8.55839	10.00117	13.00814
C	6.05200	8.40894	3.27947
H	5.33343	7.93919	2.60082
C	7.35896	8.63598	2.84053
C	8.89291	10.25886	5.91689
H	9.62756	10.82383	5.32484
H	9.47297	9.50641	6.48361
H	8.43100	10.93999	6.64506
C	5.10583	11.17849	9.05266
H	4.75035	11.66586	9.96515
C	6.38837	9.37107	8.00773
C	5.34952	8.73423	11.21679
C	7.79866	8.20739	1.47130
H	8.66538	8.78881	1.12472
H	6.99170	8.31994	0.73205
H	8.09346	7.14383	1.46802
C	6.25481	9.57298	10.53507
C	5.62804	8.36224	12.53366
H	4.92371	7.71043	13.05926
C	7.40538	10.05649	11.19380
C	7.81742	5.78012	7.95219
C	9.06811	5.30230	7.49224
C	6.86451	4.83597	8.39788
C	9.39082	3.94234	7.56160
C	7.21176	3.47667	8.45778
C	8.47217	3.03190	8.07326
H	10.36374	3.60310	7.19545
H	6.46152	2.75650	8.79493
H	8.72399	1.97076	8.14003
C	10.03434	6.22260	6.81160
C	11.18944	6.71570	7.46075
C	9.82607	6.51224	5.44472
C	12.06592	7.54353	6.75723
C	10.75216	7.31300	4.77149

C	11.86382	7.86028	5.41177
H	12.94542	7.93761	7.27490
H	10.59931	7.50699	3.70683
C	5.43594	5.16976	8.67658
C	4.63635	5.58495	7.58448
C	4.82054	4.90326	9.91466
C	3.26939	5.78735	7.77110
C	3.43921	5.09101	10.04754
C	2.64635	5.54897	9.00009
H	2.66361	6.10510	6.91700
H	2.97350	4.87620	11.01481
C	8.65045	5.95224	4.69876
H	8.81737	6.00318	3.61462
H	8.45284	4.90782	4.97805
H	7.73517	6.52359	4.91450
C	11.47137	6.37410	8.89021
H	11.43046	5.28956	9.06723
H	12.45673	6.74277	9.20564
H	10.71352	6.83290	9.54764
C	12.80461	8.77198	4.68001
H	13.83638	8.67083	5.04705
H	12.80238	8.57129	3.59878
H	12.51342	9.82737	4.81715
C	1.17936	5.80376	9.18303
H	0.60278	5.50635	8.29435
H	0.77792	5.25934	10.04980
H	0.98732	6.87768	9.34827
C	5.22406	5.72060	6.21134
H	5.86164	6.61170	6.12558
H	5.86153	4.85770	5.96989
H	4.43380	5.79262	5.45313
C	5.57549	4.42432	11.12189
H	5.57833	5.19893	11.90643
H	5.10049	3.52912	11.55274
H	6.61851	4.17585	10.89427

TS_diaryl

Sn	7.03640	8.29095	8.99921
P	9.28297	9.58002	10.00353
P	11.08277	9.05942	8.83103
P	9.30283	9.44633	7.59460
P	10.25637	11.10201	8.71360
Si	7.50756	6.01546	7.56189
C	8.86798	3.64009	8.55082
H	8.07207	3.02045	8.11478
H	9.27828	3.08393	9.41344
H	9.67578	3.72170	7.80959
C	3.77051	12.12639	8.48150
H	3.01532	12.91629	8.46624
C	6.96297	9.79350	13.58230
C	7.59867	9.45613	14.89958
H	8.40835	8.71868	14.76744
H	6.87037	9.01929	15.59850
H	8.04007	10.34424	15.37515
C	4.15670	8.48821	11.36308
H	3.36303	9.10819	10.92121
H	3.72383	7.88358	12.17177
H	4.49381	7.79448	10.57231
C	8.36594	5.01169	9.02181
C	3.48819	8.75709	5.75623
H	3.60442	8.35136	6.77142
H	3.04091	7.98018	5.12132
H	2.76823	9.58702	5.83820
C	4.79562	9.23774	5.19172
C	10.08735	6.16604	6.25265
H	10.51255	5.32658	6.82038
H	10.28584	7.09076	6.81179
H	10.65132	6.23531	5.30543
C	4.23323	11.57278	7.29485
H	3.85515	11.93794	6.33563
C	9.55727	5.79291	9.58824
H	10.32992	6.01279	8.84035
H	10.03619	5.20811	10.39412
H	9.23802	6.74576	10.03974
C	5.62641	10.09046	5.94172
C	8.17073	7.12853	5.02300
H	7.14444	7.02255	4.65383
H	8.83685	7.17584	4.14208
H	8.22626	8.10431	5.52740
C	5.19287	10.54908	7.29193
C	5.69984	5.32509	7.22848
C	5.26868	10.66756	9.70954
C	7.39353	12.32528	10.76869
H	6.67761	13.16052	10.84389
H	7.49914	12.10833	9.69573
H	8.36325	12.67484	11.14975
C	6.76512	10.66458	5.34469
C	7.11523	10.28453	4.04674
H	8.01154	10.71911	3.59368
C	7.44821	10.83747	12.79474
H	8.27738	11.44837	13.16468
C	5.17349	8.89353	3.89117
H	4.53199	8.21840	3.31615
C	6.34208	9.38749	3.30733
C	5.19389	5.80749	5.86785
H	5.29836	6.89291	5.75094
H	4.12237	5.55926	5.76280
H	5.71825	5.33112	5.02875
C	7.52158	11.76383	6.03364
H	7.00558	12.72684	5.87922
H	8.54270	11.86282	5.63995
H	7.57751	11.61475	7.11972
C	8.46431	4.65387	5.17044
H	8.77539	3.78527	5.76836
H	9.11452	4.68387	4.27703
H	7.44198	4.46868	4.81417
C	4.29929	11.67866	9.68516
H	3.97428	12.12032	10.63130
C	7.39292	4.79536	10.18786
H	7.00336	5.74872	10.58066
H	7.92315	4.29425	11.01765
H	6.53906	4.15835	9.92088
C	5.71602	10.05906	8.50873
C	4.72868	5.87140	8.28267
H	5.00789	5.61050	9.31254
H	3.71525	5.46859	8.10348
H	4.64162	6.96988	8.22186
C	5.30286	9.31797	11.85528
C	6.76550	8.95309	1.93484
H	7.25435	9.77067	1.38446
H	5.91011	8.60194	1.33981
H	7.48882	8.12215	1.99473
C	5.84886	10.33623	11.04823
C	5.87343	9.06096	13.10126

H	5.45681	8.25769	13.71644
C	8.60041	5.97687	5.93680
C	6.90089	11.13464	11.54244
C	5.63312	3.79299	7.24233
H	6.31830	3.33780	6.51254
H	4.61024	3.46899	6.97631
H	5.85844	3.36365	8.22817

Product_diaryl

Sn	7.54286	7.45163	8.68774
P	7.40564	6.73476	11.13732
P	9.55409	6.11523	10.87518
P	9.88973	7.92310	9.57398
P	9.08157	8.10219	11.66060
C	4.43078	11.29530	7.67663
H	3.71736	12.10311	7.49680
C	6.46289	9.69774	13.42869
C	6.77006	9.63523	14.89512
H	7.59320	8.92512	15.08357
H	5.90194	9.29590	15.47807
H	7.08805	10.61304	15.28604
C	4.05410	8.12557	10.93312
H	3.28241	8.77131	10.48472
H	3.58141	7.50284	11.70379
H	4.40401	7.45969	10.13085
C	4.48914	8.58840	4.49077
H	4.12460	8.44655	5.51394
H	4.23885	7.69300	3.90282
H	3.91990	9.42614	4.05549
C	5.96791	8.86268	4.42854
C	5.10617	10.70441	6.62166
H	4.95453	11.07705	5.60667
C	6.72233	9.26952	5.55395
C	6.01778	9.65162	6.81374
C	5.65596	9.85970	9.20407
C	7.90161	11.33711	10.30287
H	7.31165	11.95829	9.61229
H	8.55823	10.70359	9.68350
H	8.54841	11.99541	10.89877
C	8.08672	9.60350	5.38658
C	8.64683	9.54188	4.10942
H	9.68808	9.85030	3.98633
C	7.23133	10.48698	12.56975
H	8.03628	11.10068	12.98471
C	6.59211	8.76073	3.18320
H	6.00133	8.42400	2.32590
C	7.92815	9.11032	2.99526
C	8.96057	10.06405	6.51707
H	9.74817	10.73515	6.14675
H	9.47960	9.21110	6.98880
H	8.39620	10.58631	7.30032
C	4.72525	10.88072	8.96562
H	4.26459	11.37205	9.82639
C	6.26828	9.17573	8.12448
C	5.17779	8.93466	11.49938
C	8.58615	8.98976	1.65298
H	9.35791	9.76047	1.51088
H	7.85792	9.07311	0.83337
H	9.08432	8.00998	1.54708
C	5.99235	9.70244	10.65017
C	5.43978	8.93010	12.87194
H	4.82254	8.30413	13.52281
C	7.02489	10.49761	11.18748
C	7.69713	5.45396	7.76481
C	8.89676	4.98756	7.18165
C	6.69972	4.51524	8.10709
C	9.08509	3.61131	6.98749
C	6.91278	3.14858	7.88179
C	8.10678	2.69141	7.34012
H	10.01869	3.27353	6.53050
H	6.11824	2.44393	8.13893
H	8.26789	1.62289	7.17808
C	9.96347	5.87609	6.63723
C	11.23132	5.98915	7.24083
C	9.73941	6.45978	5.37092
C	12.18707	6.82707	6.65390
C	10.74555	7.23161	4.79227
C	11.95929	7.47307	5.44126
H	13.15381	6.94569	7.15217
H	10.58187	7.64492	3.79480
C	5.36799	4.89615	8.65587
C	4.43610	5.51973	7.79862
C	4.97232	4.48576	9.94533
C	3.14265	5.76472	8.26210
C	3.66534	4.75075	10.36597
C	2.73660	5.39171	9.54598
H	2.42204	6.23739	7.58812
H	3.36477	4.43907	11.37091
C	8.47482	6.18065	4.61340
H	8.58478	6.45425	3.55674
H	8.20300	5.11661	4.67335
H	7.62436	6.75202	5.01326
C	11.64457	5.15801	8.42123
H	12.33242	4.36272	8.08768
H	12.17874	5.76127	9.17020
H	10.79649	4.67104	8.91650
C	12.98186	8.39070	4.83770
H	13.99655	8.16177	5.19411
H	12.98019	8.32982	3.73901
H	12.77246	9.44050	5.10540
C	1.35195	5.70037	10.03388
H	0.61212	5.62765	9.22286
H	1.04659	5.01994	10.84166
H	1.29753	6.72800	10.43153
C	4.79318	5.82476	6.37352
H	5.50348	6.66049	6.28776
H	5.27564	4.95983	5.89420
H	3.89704	6.08500	5.79730
C	5.88998	3.71244	10.85052
H	5.69456	3.95493	11.90438
H	5.73728	2.62721	10.72365
H	6.94839	3.91782	10.64550

4. References

[1] a) N. Kuhn, T. Kratz, Synthesis 1993, 1993, 561-562; bR. S. Simons, L. Pu, M. M. Olmstead, P. P. Power, Organometallics 1997, 16, 1920-1925.

[2] a) A. E. Seitz, M. Eckhardt, A. Erlebach, E. V. Peresypkina, M. Sierka, M. Scheer, J. Am. Chem. Soc. 2016, 138, 10433-10436; b) R. Yadav, T. Simler, S. Reichl, B. Goswami, C. Schoo, R. Köppe, M. Scheer, P. W. Roesky, J. Am. Chem. Soc. 2020, 142, 1190-1195.

[3] APEX suite of crystallographic software, APEX 3, Version 2016.9-0, Bruker AXS Inc., Madison, Wisconsin, USA, 2016.

[4] CrysAlisPro, Version 1.171.39.46, Rigaku Oxford Diffraction, Abingdon, Oxfordshire, England, 2018.

[5] SAINT,Version 8.37A, andSADABS, Version 2016/2, Bruker AXS Inc., Madison, Wisconsin, USA,2016.

[6] G. M. Sheldrick, Acta Crystallogr. Sect. A2015, 71, 3–8.

[7] G. M. Sheldrick, Acta Crystallogr. Sect. C2015, 71, 3–8.

[8] C. B. Hübschle, G. M. Sheldrick, B. Dittrich, J. Appl. Cryst.2011, 44, 1281–1284

[9] International Tables for Crystallography, Vol. C (Ed.: A. J. Wilson), Kluwer Academic Publishers, Dordrecht, The Netherlands, 1992, Tables 6.1.1.4 (pp. 500–502), 4.2.6.8 (pp. 219–222), and 4.2.4.2 (pp. 193–199)

[10] F. Neese, Wiley Interdiscip. Rev. Comput. Mol. Sci. 2018, 8, e1327.

[11] a) F. Neese, F. Wennmohs, A. Hansen, U. Becker, Chem. Phys. 2009, 356, 98-109; b) R. Izsak, F. Neese, J. Chem. Phys. 2011, 135, 144105; c) F. Weigend, J. Comput. Chem. 2008, 29, 167-175.

[12] a) J. P. Perdew, M. Emzerhof, K. Burke, J. Chem. Phys. 1996, 105, 9982-9985; b) C. Adamo, V. Barone, J. Chem. Phys. 1999, 110, 6158-6170; c) F. Weigend, R. Ahlrichs, Phys. Chem. Chem. Phys. 2005, 7, 3297-3305.

[13] B. Metz, H. Stoll, M. Dolg, J. Chem. Phys. 2000, 113, 2563-2569.

[14] a) S. Grimme, J. Antony, S. Ehrlich, H. Krieg, J. Chem. Phys. 2010, 132, 154104; b) S. Grimme, S. Ehrlich, L. Goerigk, J. Comput. Chem. 2011, 32, 1456-1465.

[15] F. Weigend, R. Ahlrichs, Phys. Chem. Chem. Phys. 2005, 7, 3297-3305.

[16] a) M. Saitow, U. Becker, C. Riplinger, E. F. Valeev, F. Neese, J. Chem. Phys. 2017, 146, 164105; b) C. Riplinger, F. Neese, J. Chem. Phys. 2013, 138, 034106; c) Y. Guo, C. Riplinger, U. Becker, D. G. Liakos, Y. Minenkov, L. Cavallo, F. Neese, J. Chem. Phys. 2018, 148, 011101; d) D. G. Liakos, F. Neese, J. Chem. Theor. Comput. 2015, 11, 4054-4063.

[17] A. Hellweg, C. Hättig, S. Höfener, W. Klopper, Theor Chem Acc 2007, 117, 587-597.

[18] a) A. V. Marenich, C. J. Cramer, D. G. Truhlar, J. Phys. Chem. B 2009, 113, 6378-6396; b) C. J. Cramer, D. G. Truhlar, Acc. Chem. Res. 2008, 41, 760-768.

[19] Caldararu, O.; Olsson, M. A.; Riplinger, C.; Nesse, F.; Ryde, U. J. Comput. Aided. Mol. Des. 2017, 31, 87-106.

[20] M. D. Hanwell, D. E. Curtis, D. C. Lonie, T. Vandermeersch, E. Zurek, G. R. Hutchison, J. Cheminformatics 2012, 4, 17.

[21] G. Knizia, J. Chem. Theory Comput. 2013, 9, 4834-4843.

[22] C. van Wüllen, J. Chem. Phys. 1998, 109, 392-399.

[23] a) M. Reiher, WIREs Comput. Mol. Sci. 2012, 2, 139-149; b) A. Wolf, M. Reiher, B. A. Hess, J. Chem. Phys. 2002, 117, 9215-9226.

[24] a) J. Vícha, J. Novotný, S. Komorovsky, M. Straka, M. Kaupp, R. Marek, Chem. Rev. 2020, 120, 7065-7103; b) F. Alkan, S. T. Holmes, R. J. Iuliucci, K. T. Mueller, C. Dybowski, Phys. Chem. Chem. Phys. 2016, 18, 18914-18922; c) T. B. Demissie, J. Chem. Phys. 2017, 147, 174301.
